# Promoting and countering misinformation during Australia’s 2019–2020 bushfires: a case study of polarisation

**DOI:** 10.1007/s13278-022-00892-x

**Published:** 2022-06-24

**Authors:** Derek Weber, Lucia Falzon, Lewis Mitchell, Mehwish Nasim

**Affiliations:** 1 School of Computer Science, University of Adelaide, North Terrace, Adelaide, SA 5005 Australia; 2 Defence Science and Technology Group, West Terrace, Edinburgh, SA 5111 Australia; 3 School of Psychological Sciences, University of Melbourne, Parkville Campus, Melbourne, VIC 3010 Australia; 4 School of Mathematical Sciences, University of Adelaide, North Terrace, Adelaide, SA 5005 Australia; 5 ARC Centre of Excellence for Mathematical and Statistical Frontiers, North Terrace, Adelaide, SA 5005 Australia; 6 College of Science and Engineering, Flinders University, South Road, Tonsley, SA 5042 Australia; 7 Department of Computer Science & Software Engineering, University of Western Australia, Crawley, Australia

**Keywords:** Social media, Information campaigns, Polarisation, Misinformation, Crisis, Twitter

## Abstract

During Australia’s unprecedented bushfires in 2019–2020, misinformation blaming arson surfaced on Twitter using #ArsonEmergency. The extent to which bots and trolls were responsible for disseminating and amplifying this misinformation has received media scrutiny and academic research. Here, we study Twitter communities spreading this misinformation during the newsworthy event, and investigate the role of online communities using a natural experiment approach—before and after reporting of bots promoting the hashtag was broadcast by the mainstream media. Few bots were found, but the most bot-like accounts were *social bots*, which present as genuine humans, and trolling behaviour was evident. Further, we distilled meaningful quantitative differences between two polarised communities in the Twitter discussion, resulting in the following insights. First, *Supporters* of the arson narrative promoted misinformation by engaging others directly with replies and mentions using hashtags and links to external sources. In response, *Opposers* retweeted fact-based articles and official information. Second, Supporters were embedded throughout their interaction networks, but Opposers obtained high centrality more efficiently despite their peripheral positions. By the last phase, Opposers and unaffiliated accounts appeared to coordinate, potentially reaching a broader audience. Finally, the introduction of the bot report changed the discussion dynamic: Opposers only responded immediately, while Supporters countered strongly for days, but new unaffiliated accounts drawn into the discussion shifted the dominant narrative from arson misinformation to factual and official information. This foiled Supporters’ efforts, highlighting the value of exposing misinformation. We speculate that the communication strategies observed here could inform counter-strategies in other misinformation-related discussions.

**Supplementary Information:**

The online version contains supplementary material available at 10.1007/s13278-022-00892-x.

## Introduction

People share an abundance of useful information on social media during crises (Bruns and Liang 2012; Bruns and Burgess 2012). This information, if analysed correctly, can rapidly reveal newsworthy events such as imminent civil unrest, natural disasters or accidents (Tuke et al. 2020). Not all content is helpful, however: different entities may try to popularise false narratives using sophisticated social bots and/or engaging humans. The spread of such misinformation not only makes it difficult for analysts to use Twitter data for public benefit (Nasim et al. 2018) but may also encourage large numbers of people to adopt the false narratives causing social disruption and polarisation, which may then influence public policy and action, and thus can be particularly destabilising during crises (Singer and Brooking 2019; Kušen and Strembeck 2020; The Soufan Center 2021; Scott 2021).

In our previous work (Weber et al. 2020), we identified two polarised communities in a misinformation-laden Twitter discussion regarding Australia’s ‘Black Summer’ 2019/2020 bushfires using the hashtag #ArsonEmergency. Using an exploratory mixed-method approach applied to temporal phases of the discussion, our analyses identified differences in behaviour and content between the *Supporters* of the ‘arson narrative’ and the *Opposers* who countered it with fact check articles and official information. This analysis also included the context of the broader discussion by participants *Unaffiliated* with either polarised group. The first of three phases ended with the publication of a ZDNet article reporting preliminary research on the discussion, which revealed anomalous levels of bot activity (Stilgherrian 2020).

In the interests of exploring the potential effects of interventions to counter misinformation, we now extend this work using a natural experiment lens by treating the publication of the ZDNet article as if it were a deliberate intervention. In this way, Phase 1 ends at the point of the intervention. We find that the intervention was successful at changing the dominant content being shared in the discussion, drew significant attention by reaching out to the mainstream media (MSM), but also inflamed the debate by drawing in more Supporters, though their response was short-lived. Further, we expand the results offered by the original researchers, whose own follow-up research concluded that the role of trolls was significant (Graham and Keller 2020), that the activities may have been part of a larger disinformation campaign (Keller et al. 2020).

This paper exemplifies the use of state-of-the-art techniques to interrogate the activities of misinformation-promoting and -opposing groups within the context of the broader discussion, incorporating network science, statistics and content analyses, with particular attention paid to coordinated and inauthentic behaviour. The role of researchers in countering misinformation with both non-peer-reviewed and peer reviewed communications is also considered, along with other opportunities to generalise the work to address other misinformation-heavy discussions.

### The ‘Black Summer’ bushfires and misinformation on Twitter

The 2020 Australian ‘Black Summer’ bushfires (a.k.a., wildfires) burnt over 16 million hectares, destroyed over 3500 homes, and caused at least 33 human and a billion animal fatalities,^1^ and attracted global media attention. During the bushfires, as in other crises, social media provided a mechanism for people in the fire zones to provide on-the-ground reports of what was happening around them, a way for those outside to get insight into the events as they occurred (including authorities and media), but also a way for the broader community to connect and process the imagery and experiences through discussion. The lack of the traditional information mediator or gatekeeper role played by the mainstream media on social media permits factual errors, mis-interpretation and outright bias to proliferate without check in a way it could not in decades past. Our previous analysis of online discussion at this time (Weber et al. 2020) showed:

- The bushfires were a topic of much discussion on Twitter, which influenced media coverage.
- The Supporter and Opposer communities differed significantly in their interpretation of the ongoing events.
- False narratives and misinformation present in the discussion, which we label the ‘arson narrative’, was promoted primarily by Supporters, and included that:
  - the bushfires were mostly caused by arson;
  - preventative backburning efforts had been reduced due to green activism (previously presented in 2009^2^);
  - Australia commonly experiences such bushfires (previously put forward in 2013^3^); and
  - climate change is not related to bushfires.

In Weber et al. (2020), we collected 18 days of Twitter data to examine the discussion on #ArsonEmergency and replicate findings reported in ZDNet (Stilgherrian 2020). In the process, analyses revealed two clearly polarised groups in the retweet network, distinctly different in behaviour and content. *Supporters* promoted the narrative that Australia’s ‘Black Summer’ bushfires were primarily caused by arson, relying on misinformation and biased reporting, while the *Opposers* countered with official announcements and fact-check articles. We determined that the Opposers responded to the growing Supporter and Unaffiliated account activity on #ArsonEmergency, when it was exposed in a ZDNet article (Stilgherrian 2020). The publication of the ZDNet article was the trigger that drew the attention of the mainstream media, which promulgated the exposé further, drawing in many more Unaffiliated accounts and noticeably changed the nature of the discussion. Using a different bot detection system, many fewer bots were found than in the analysis reported in the ZDNet article.

The analyses did not explore whether the polarisation in the retweet network was also reflected in networks made from other interactions, nor how the groups responded to each others’ behaviour beyond the initial Opposer response, nor any exploration of growth patterns of the groups, in case they artificially grew their numbers with external help (e.g. from beyond national borders). Questions remain about the communication strategies used, including whether there is any evidence of coordinated amplification (Weber and Neumann 2021), which may suggest the presence of an organised misinformation or disinformation campaign. Although evidence of bots was found to be limited, later work by Graham and Keller (2020) and Keller et al. (2020) clarified the significant role of trolls in the discussion, arguing that it amounted to participation in a broader disinformation campaign, thus exploration of trolling and similar inauthentic behaviours (Gleicher 2018) remain to be explored. Although we do not consider the intention of those promulgating false narratives in the data, whether the actions constitute an (unintentional) misinformation or a (deliberate) disinformation campaign, our analyses can shed light on the presence and activity of trolls and other inauthentic accounts.

We present a mixed-method analysis of the Twitter activity using the term ‘ArsonEmergency’ approximately a week before and after the publication of the ZDNet article (Stilgherrian 2020), which we treat as an ‘intervention’ to counter misinformation in the discussion. We make use of a combination of complementary social network analyses (SNA), behavioural and content analyses pivoted around the intervention, the polarised groups and unaffiliated discussion participants. Analysis of the networks of different interactions in the data reveal how central these groups became and to what degree they connected to each other and the broader discussion. We also consider patterns of inauthentic behaviour by presenting and demonstrating a simple method for exposing inauthentic tweets by their text patterns.

### Related work

The study of Twitter during crises and times of political significance is well-established (Bruns and Liang 2012; Bruns and Burgess 2012; Flew et al. 2014; Marozzo and Bessi 2017; Graham et al. 2020), and has provided recommendations to governments and social media platforms alike regarding its exploitation for timely community outreach. The social media response of the Australian Queensland State Government was praised for its use of social media to manage communication during devastating floods (Bruns and Burgess 2012), and analyses of coordinated behaviour have revealed significant organised anti-lockdown behaviour during the COVID pandemic (Graham et al. 2020; Magelinski and Carley 2020; Loucaides et al. 2021) and in the lead up to the January 6 Capitol Riots in America (Scott 2021; Ng et al 2021). The continual presence of trolling and bot behaviour diverts attention and can confuse the public at times of political significance, whether it is to generate artificial support for policies and their proponents (Keller et al. 2017; Rizoiu et al. 2018; Woolley and Guilbeault 2018), harass opponents (Keller et al. 2017; CREST 2017) or just pollute existing communication channels (Woolley 2016; Nasim et al. 2018; Kušen and Strembeck 2020). Malign actors can also foster online community-based conflict (Kumar et al. 2018; Datta and Adar 2019; Mariconti et al. 2019) and polarisation (Conover et al. 2011; Garimella et al. 2018; Morstatter et al. 2018; Villa et al. 2021).

Studies of the spread of rumours and counter-efforts on Twitter during crises, and people’s response to them, have also confirmed the importance of authorities injecting reliable clarifications into the information space. Wang and Zhuang (2018) studied how Twitter users responded when they encountered misinformation in a number of different discussions. They developed a decision tree that modelled a variety of user behaviours including initially attempting to confirm a rumour, simply doubt it or disseminate it, and then also behaviour after their disseminated post had been debunked in a reply. Populating the decision tree with real-world data, they found that questions regarding rumours were frequently answered, but of the vast majority of people who disseminated the rumours in question (via retweet), fewer than 20% of users would take some remedial action if their retweet was debunked. The decision tree concept was extended by Agarwal et al. (2022) using a game-theoretic approach, creating decision models that examined how authorities can select which rumours to debunk or clarify, and how important timeliness is compared with the quality of the clarification. Using several datasets relating to true and false rumours, they showed the value of authorities providing clarifying information, which dampened the dissemination of false rumours, as well as that, up to a point, it is better for authorities to delay debunking a false rumour until high quality information is available. Hunt et al (2020) examined the cross-platform information sharing behaviour of Twitter users during Hurricanes Harvey and Irma, which both affected America in September 2017. Although most retweets were by non-verified accounts, 90% of verified account retweets were of factual reports from government agencies. The most frequently mentioned URLs and URL domains belonged to government agencies and news media, and most referred to easily accessible public websites, rather than posts on other social media sites, similar to previous findings regarding EXTERNAL URLs (Weber et al. 2020). They also found that posts debunking rumours can have a continued effect: they observed that a given rumour lasted only a third as long during the second hurricane, as the debunking information was still present in the online discussion. Thus, there is significant value in government agencies having an active social media presence during crises, as a source for credible and correct information.

Misinformation can be regarded as the sharing of unintentionally false information, while disinformation is shared knowing that it is false or biased in a deliberate attempt to deceive others (Wardle and Derakhshan 2017). Information campaigns rely on groups of accounts disseminating a particular narrative as propaganda, using truthful information, true information with a biased presentation, or outright falsehoods (Kavanagh and Rich 2018). The blending of true and false information makes it harder to identify (Starbird 2019) and easier to convince others to share, thereby making modern information operations ‘participatory’ in nature (Starbird et al. 2019). Due to the degree to which social media is enmeshed in modern life, misinformation on social media is a topic of much study (Kumar and Shah 2018; Starbird 2019; Singer and Brooking 2019; Graham et al. 2020), with growing attention to its overall effect on society (Starbird 2019; Carley 2020), but many relevant current events are yet to be explored in the peer-reviewed literature. Instead, researchers have turned to other methods to quickly warn of the dangers of misinformation via other channels; examples include Graham and Keller ’s interview with the technology magazine ZDNet (Stilgherrian 2020) and their follow-up article on The Conversation (Graham and Keller 2020), a publisher of ‘research-based news and analysis’,^4^ while commissioned reports provide an opportunity to present more comprehensive yet still not peer-reviewed analyses (e.g. Wardle and Derakhshan 2017; Graham et al. 2020; Smith et al. 2020). Because social media has become such a mainstay of modern communication, misinformation on social media is often amplified on the mainstream media (MSM), or by prominent individuals, often when it aligns with their ideological outlook, which then feeds back into social media as people discuss it further; such cycles have been known to be deliberately fostered (Benkler et al 2018; Starbird and Wilson 2020; Badham 2021). Patterns of fire-related misinformation similar to those observed on #ArsonEmergency were repeated in the US during Californian wildfires in mid-2020, even causing armed vigilante gangs to form to counter non-existent Antifa activists who were blamed for the fires on social media.^5^ Arson was again blamed for the 2021 fires around the Mediterranean, throughout southern Europe and in northern Africa,^6^ even as the United Nations’ Intergovernmental Panel on Climate Change released its sixth Assessment Report stating that humans’ effect on climate is now ‘unequivocal’ (IPCC In Press). Furthermore, when the misinformation involved relates to conspiracy theories involving public health measures during a global pandemic, the risk is that adherents will turn away from other evidence-based policies, as we see with vaccine hesitancy (Ball and Maxmen 2020), adoption of flat earth beliefs (Brazil 2020) and other conspiratorial anti-government sentiments (The Soufan Center 2021).

Online conversation dynamics can produce information environments vulnerable to misinformation, such as *echo chambers* and *filter bubbles* (Pariser 2012; Bruns 2019). While studying COVID-19 vaccine-related narratives in mid-2020, Smith et al. (2020) highlight two primary ‘market failures of the information industry: data deficits and data oversupply’ (p.20, Smith et al. 2020). These both relate to the amount of information, particularly credible information, in a discussion relative to the demand. A *data oversupply* results in a crowded information space, where people are easily confused and overloaded by (sometimes contradictory) information, which causes them to disengage. A *data deficit*, in contrast, occurs when there is a lack of credible information about an issue but significant demand for it. Although data deficits are not deliberately created (experts may not realise what information people require or which require it), it can be vulnerable to the introduction of misinformation and exploited with disinformation. In our study, it is arguable that the ’data deficit’ of #ArsonEmergency was, in fact, created deliberately ( Graham and Keller 2020, argue this), thereby establishing a *data void* into which disinformation based on the arson narrative could be allowed to flourish before linking it to broader discussions.

Studies of virality of social media content also examine similar natural experiments. The question of predictability of retweet cascades has been explored. Cheng et al. (2014) examined how to predict the magnitude of a cascade of retweets or Facebook shares, finding that its breadth (the maximum frequency of shares within a certain period after the original post) is a strong indicator of the final overall number of shares. Rizoiu et al. (2018) proposed a point process model of retweet cascades to measure how influential accounts are in their exploration of social bot manipulation of Twitter during the 1st 2016 US presidential debate. Other studies have examined how the ‘buzz’ of Facebook posts can predict whether they can go viral (Deusser et al. 2018), and then also the nature of these ‘buzzes’ as ‘firestorms’ of anger, ‘lovestorms’ filled with messages of affection and support or ‘hot topics’ of enthusiasm (Jansen 2019). Strathern et al. (2020) explored the triggers behind firestorms, particularly those based on moral infractions. Conclusions from these studies can inform communication strategies to respond to bad press or other events of unwanted attention. In this study, it can be argued that the intervention was aimed at exposing a previously obscured discussion to generate a response to it, rather than to quell a response.

Finally, the natural experiment model has been used in the study of online discussion dynamics. Wang et al. (2018) studied the effect of user verification on the spread of fake news on Weibo, finding that fake news is dampened when people know they are discoverable (via verification with the platform), but this effect is outweighed by greater engagement if the verification is made public (e.g. with a badge icon) due to the credibility it implies. A study of which EU politicians benefit most from bot followers on Twitter examined the drop in follower numbers after Twitter’s July 2018 purge of bot accounts (Silva and Proksch 2020). Results showed the radical right lost the most. Given political parties rely on being ‘popular’ and the media’s use of Twitter to judge this, it suggests they received the most benefit, as it drew unwarranted attention to their causes. Here, our natural experiment model allows us to examine the change in dynamics of an online discussion before and after the ZDNet article’s publication.

### Research questions

We use the following research questions to guide our exploration of the effects of the ZDNet article intervention on the spread of misinformation and on the behaviour of participants in the #ArsonEmergency discussion:

**RQ1** How did the behaviour of those promoting misinformation differ from those countering it, and did behaviour change as a result of the intervention of the ZDNet article?

**RQ2** How central were the Supporter and Opposer communities to the discussion and how insular were they from each other and the broader discussion?

**RQ3** How did the Supporter and Opposer communities make use of retweets, hashtags and URLs to promote their narrative? What evidence is there of coordination?

**RQ4** What role did inauthentic behaviour play?

The aim of these research questions is to provide guidelines to exploring a communication space in which it is known there is some kind of misinformation campaign using a natural experiment approach. They encourage identification of the communities promoting and potentially countering the misinformation, differences in their behaviour and communication strategies, and to what extent inauthentic behaviour (such as use of automation and trolling) is exploited. Furthermore, based on this context, they direct the examination to consider any changes before and after the intervention and how they relate to the intervention.

In the remainder of this paper, we describe our mixed-method analysis and the datasets used. We briefly recap the timeline analysis (Weber et al. 2020), before examining the growth of the discussion, comparing it to other contemporary related and unrelated contentious hashtag-based discussions. We address our research questions and discuss how our findings can be generalised to analyse and counter misinformation elsewhere before concluding with thoughts for future work.

## The data and its timeline

The primary dataset was collected over an 18 day period at the height of the bushfires using the term ‘ArsonEmergency’ (see Table 1). For comparison, over the same time period, a second bushfire-related dataset was collected using the search term ‘AustraliaFire’, along with a non-bushfire-related dataset focused on #Brexit.

**Table 1:** The datasets were collected from 31 December 2019 to 17 January 2020

Dataset	Tweets	Accounts	Collection method
*Primary*			
ArsonEmergency	27,546	12,872	Twarc^a^ searches on 8, 12, and 17 January
*Comparison*			
AustraliaFire	111,966	96,502	Twarc searches on 8 and 17 January
#Brexit	187,792	78,216	Streamed with RAPID (Lim et al. 2018)

Both Twarc and RAPID communicate with Twitter’s standard Application Programming Interfaces (APIs)

^a^https://github.com/DocNow/twarc

Broader searches using multiple related terms were not conducted due to time constraints and the original study’s aim of comparison with Graham and Keller’s findings (Graham and Keller 2020). Due to the use of Twint^7^ in that study, differences in dataset were likely but expected to be minimal. Differences in datasets collected simultaneously with different tools have been previously noted (Weber et al. 2021). Live filtering was also not employed for the bushfire-related searches, as the research started after Graham and Keller’s findings was reported.

Twitter may have removed inauthentic content in the time between it being posted and us conducting searches as part of data cleaning routines. For these reasons, some of the content observed by Graham and Keller was expected to be missing from our dataset. This lack of consistency between social media datasets for comparative analyses is a growing challenge recently identified in the benchmarking literature (Assenmacher et al 2021).

Tweets by Graham and Keller, whose research was referred to in the ZDNet article (Stilgherrian 2020) were not removed from the ‘ArsonEmergency’ dataset, as it was felt their effect was limited. Graham and Keller posted six and three retweets, respectively, all after the ZDNet article was published. As Graham and Keller were mentioned in tweets promoting the ZDNet article and, three days later, the Conversation article by them (Graham and Keller 2020), their Twitter handles appeared in 106 and 8 tweets posted between the 7th and the 11th of January, peaking on the days the articles were published.

An inspection for meta-discussion (uses of the hashtags without the ‘#’ symbol, avoiding contributing to the hashtag discussion) in the ‘ArsonEmergency’ and ‘AustraliaFire’ datasets revealed very few uses (34 tweets and 100 tweets, respectively), but all uses followed the ‘intervention’ point, and so could be considered responses to the media reports. Manual examination of the tweets confirmed this.

### The timeline

This study focuses on about a week of Twitter activity before and after the publication of the ZDNet article (Stilgherrian 2020), which effectively acted as an intervention to counter the misinformation in the discussion. The ZDnet article was published at 6:03am GMT (5:03pm AEST^8^) on 7 January 2020, and was then reported more widely in the MSM morning news, starting around 13 h later. We use the same temporal markers as Weber et al. (2020) to define three phases:

- *Phase 1*: Before 6am GMT, 7 January 2020;
- *Phase 2*: From 6am to 7pm GMT, 7 January 2020; and
- *Phase 3*: After 7pm GMT, 7 January 2020.

Our primary interest, however, lies in the differences before and after Phase 1 ends.

**Fig. 1:**
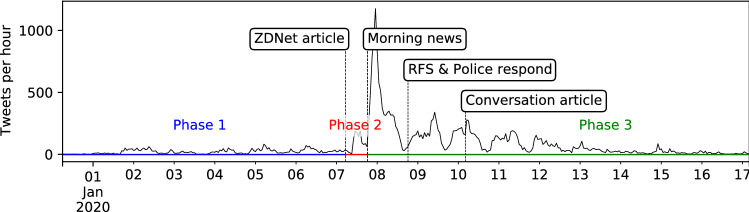
Hourly tweet activity in the ‘ArsonEmergency’ dataset, annotated with the phases, noting their significance in real-world terms

Figure 1 shows the number of tweets posted each hour in the ‘ArsonEmergency’ dataset, and highlights the phases and notable events including: the publication of the ZDNet article; when the story hit the MSM; the time at which the Rural Fire Service (RFS) and Victorian Police countered the narratives promoted on the #ArsonEmergency hashtag; the publication of a Conversation article clarifying the ZDNet findings (Graham and Keller 2020); and the clear subsequent diurnal cycle.

Since late September 2020, Australian and international media had reported on the bushfires around Australia, including stories and photos drawn directly from social media, as those caught in the fires shared their experiences. No one hashtag had emerged to dominate the online conversation and many were in use, including #AustraliaFires, #ClimateEmergency, #bushfires and #AustraliaIsBurning.

The ZDNet article publication generated an immediate response, with over 1, 200 tweets in Phase 2 by 927 accounts, 824 of which had not tweeted in Phase 1. Nearly 85% of the Phase 2 tweets were retweets, and 60% of those promoted the ZDNet article, including 357 retweets of a single tweet mentioning it. Most Phase 2 activity occurred in the Australian evening period, and the diurnal cycle visible in the time series indicates the majority of activity was local to Australia (or at least to its timezones). The biggest reaction came immediately after the story reached the morning news the next day, while the publication of the Conversation article appears not to have generated a significant response.

### Growth of the discussions

**Fig. 2:**
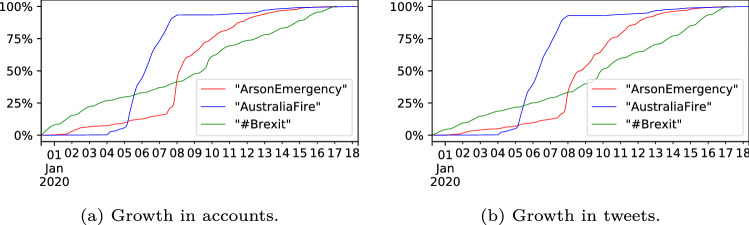
The growth of the ArsonEmergency, AustraliaFire and #Brexit datasets in terms of accounts joining the discussion and the tweets posted

To consider if the pattern of discussion growth in ‘ArsonEmergency’ is typical, we compared the discussion with two other contemporary discussions in terms of user growth (i.e. number of new accounts joining the discussion) and tweet growth (Fig. 2). The similarity in the user and tweet growth lines indicates that as new accounts joined each discussion, they usually only posted a single tweet. The #Brexit discussion lacks a clear intervention event and so its growth is smooth and consistent.^9^ In contrast, ‘AustraliaFire’ discussion appears to be a hashtag campaign instigated by people in Pakistan and Germany resulting in 45k retweets. Many of the retweeting accounts were suspended, so it is possible they were driven by botnets, and the campaign stops growing suddenly after a few days. The ‘ArsonEmergency’ dataset’s growth pattern clearly shows the point of the intervention, but it continues to grow for several more days after the initial response.

## Polarised communities

**Fig. 3:**
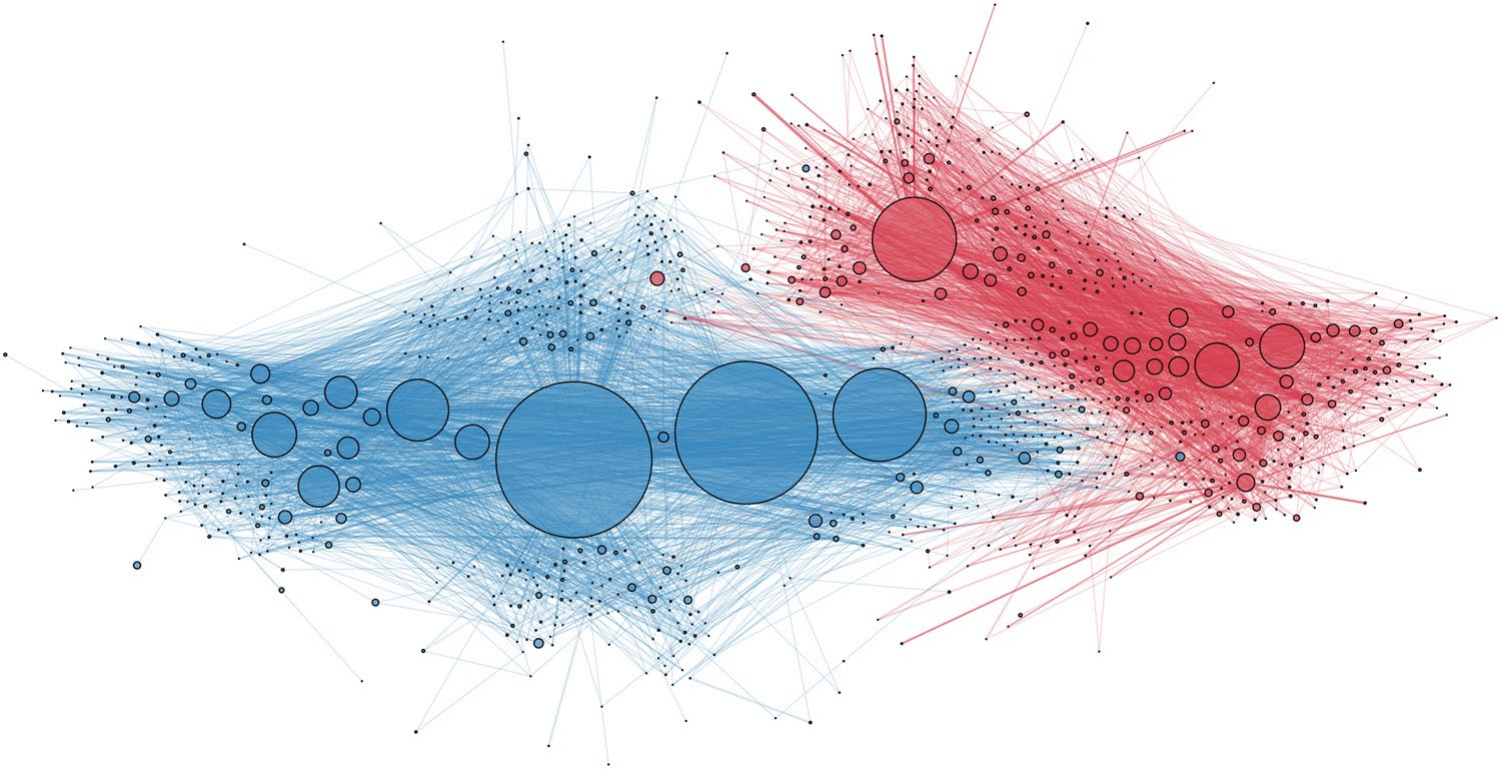
Retweet network of the #ArsonEmergency discussion showing clear polarisation, reproduced from Figure 3 in Weber et al. (2020). On the left in blue is the *Opposer* community, which countered the arson narrative promoted by the red *Supporter* community on the right. Nodes represent users. An edge from one node to another means that the account represented by the first node retweeted one of tweets of the account represented by the second. Node size corresponds to indegree centrality, indicating how often the account was retweeted

Our original study was aimed at identifying information campaigns and characterising the behaviour and content of the groups behind them (Weber et al. 2020). Given retweets are the most obvious manner in which to endorse content on Twitter, we examined the ‘ArsonEmergency’ retweet network, finding the largest component dominated by two large and apparently polarised communities (Fig. 3). Although retweets are not necessarily direct endorsements, work by Metaxas et al. (2015) and Falzon et al. (2017) found that retweeting is used to indicate trust in the content of a tweet or its source, or to help form social bonds (through reciprocal retweeting), and so retweets can imply agreement or likemindedness, at the very least. Based on manual examination of the ten most retweeted accounts in each community, we labelled one Supporters of the arson narrative and the other Opposers (shown as the 497 red and 593 blue nodes, respectively, in Fig. 3). The flow of information between the communities was limited and reflected dissemination and re-dissemination of the same narrative within each community. Such closed communities may develop into *echo chambers*, communities within which only acceptable opinions on a matter may be shared, or even *filter bubbles*, communities that explicitly prevent new opinions and information from being entertained (Pariser 2012; Bruns 2019). Retweets appear to coalesce within these communities, emphasising the echo chamber affect, as has been observed in Facebook comment dynamics (Nasim et al. 2013).

These two groups, Supporters and Opposers, and those users Unaffiliated with either group, are used to frame the remainder of the analysis in this paper.

### Community timelines

**Fig. 4:**
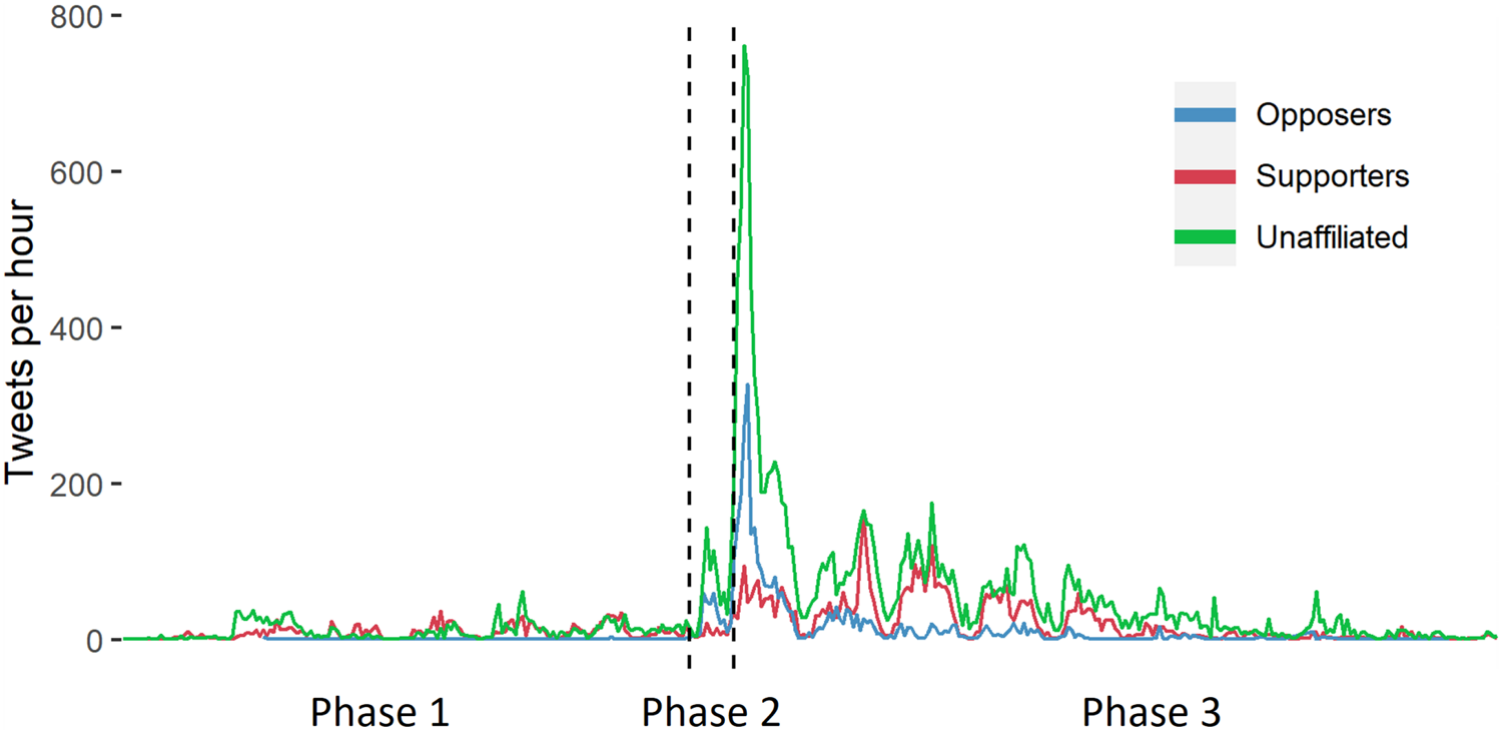
A timeline of each communities’ activity over the collection period

The relative behaviour of the communities over the collection period, shown in Fig. 4, informs several key observations. The first is the impact of the story reaching the MSM: the peaks of both Opposer and Unaffiliated contributions are on the morning of Phase 3, immediately after the story appeared on the morning bulletins. Despite the much greater number of Unaffiliated accounts (11,782), their peak is only a little more than twice that of the 593 Opposer accounts. Unaffiliated and Supporter accounts are active during the entire collection, but Supporters’ activity is prominent each day in Phase 3, and peaks on the second day of Phase 3. That peak might have occurred as a response to the previous peak, as by that time the news would have had a full day to disseminate around the world. By reaching a broader audience via the MSM, more Supporter accounts may have been drawn into the online discussion. Supporter activity increased dramatically in Phase 3, potentially in response to the Opposer pushback in Phase 2.

**Fig. 5:**
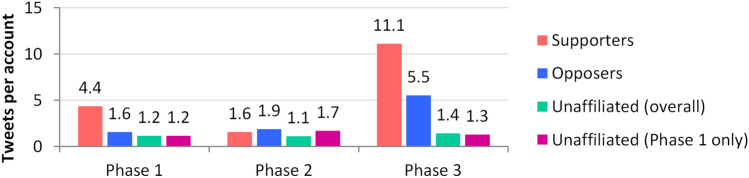
Tweets per account in each phase for the Supporters, Opposers, Unaffiliated accounts overall and Unaffiliated accounts active in Phase 1

Analysis confirms that the composition of the Unaffiliated participants did change across the phases. Few of the 1680 Unaffiliated accounts active in Phase 1 appeared in the later phases (30 in Phase 2 and 427 in Phase 3), and their contributions were not significantly higher than the new Unaffiliated accounts (Fig. 5). It is clear that Supporters and Opposers’ contributions changed dramatically, however. Supporters were the most active in the larger phases, particularly in the last. Opposers were most active in the final phase also, but contributed at a rate of less than half that of Supporters, though they did contribute slightly more than Supporters per account in Phase 2, immediately after the release of the ZDNet article.

**Fig. 6:**
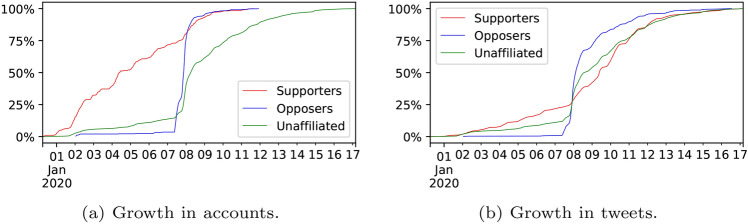
The growth of the ArsonEmergency discussion in terms of accounts joining the discussion and the tweets they posted

Examining the accumulation of new accounts (Fig. 6a) and new tweets (Fig. 6b) shows that #ArsonEmergency was steadily accruing Supporters until the ZDNet article (Stilgherrian 2020), at which point the community was established and remained active for several days into Phase 3. The Opposer community joined almost entirely in Phase 2, and its activity was mostly confined to that phase, while the Unaffiliated continued to join the discussion well into Phase 3. The publication of the ZDNet article appears to have drawn in large numbers of Opposers and Unaffiliated, while the Supporter growth immediately plateaued.

Finally, a clear diurnal effect can be in Fig. 4 with daily peaks of activity occurring during Australian daytime hours, implying that the majority of the activity is domestic. Analysis of the ‘lang’ field in the tweets^10^ confirmed that over 99% of tweets used ‘en’ (English, 90.5%) or ‘und’ (undefined, 8.7%). An analysis of self-reported locations indicated that most accounts were Australian (88% of Opposers, 77% of Supporters and 72% of Unaffiliated), while foreign accounts were most often from the US or UK.

### Behaviour

User behaviour on Twitter can be examined through the features used to connect with others and through content. Here, we consider how active the different groups were across the phases of the collection, and then how that activity manifested itself in the use of mentions, hashtags, URLs, replies, quotes and retweets. The statistics in Table 2 are a superset of those discussed in our previous work (Weber et al. 2020), but serve to inform the later analyses in this paper.

**Table 2:** Activity of the polarised retweeting accounts, by interaction type in phases

Group	Tweets	Accounts	Hashtags	Mentions	Quotes	Replies	Retweets	URLs
Phase 1								
Supporters								
*Raw count*	1573	360	2257	1020	185	356	938	405
*Per account*	4.37	–	6.27	2.83	0.51	0.99	2.61	1.13
*Per tweet*	–	–	1.43	0.65	0.12	0.23	0.60	0.26
Opposers								
*Raw count*	33	21	100	5	8	2	20	9
*Per account*	1.57	–	4.76	0.24	0.38	0.10	0.95	0.43
*Per tweet*	–	–	3.03	0.15	0.24	0.06	0.61	0.27
Phase 2								
Supporters								
*Raw count*	121	77	226	64	11	29	74	24
*Per account*	1.57	–	2.94	0.83	0.14	0.38	0.96	0.31
*Per tweet*	–	–	1.87	0.53	0.09	0.24	0.61	0.20
Opposers								
*Raw count*	327	172	266	34	7	14	288	31
*Per account*	1.90	–	1.55	0.20	0.04	0.08	1.67	0.18
*Per tweet*	–	–	0.81	0.10	0.02	0.04	0.88	0.09
Phase 3								
Supporters								
*Raw count*	5278	474	7414	2685	593	1159	3212	936
*Per account*	11.14	–	15.64	5.66	1.25	2.45	6.78	1.97
*Per tweet*	–	–	1.40	0.51	0.11	0.22	0.61	0.18
Opposers								
*Raw count*	3227	585	3997	243	124	95	2876	359
*Per account*	5.52	–	6.83	0.42	0.21	0.16	4.92	0.61
*Per tweet*	–	–	1.24	0.08	0.04	0.03	0.89	0.11
Overall								
Supporters								
*Raw count*	6972	497	9897	3769	789	1544	4224	1365
*Per account*	14.03	–	19.91	7.58	1.59	3.11	8.50	2.75
*Per tweet*	–	–	1.42	0.54	0.11	0.22	0.61	0.20
Opposers								
*Raw count*	3587	593	4363	282	139	111	3184	399
*Per account*	6.05	–	7.36	0.48	0.23	0.19	5.37	0.67
*Per tweet*	–	–	1.22	0.08	0.04	0.03	0.89	0.11
Unaffiliated								
*Raw count*	16,987	11,782	22,192	3474	615	1377	14,119	1790
*Per account*	1.44	–	1.88	0.29	0.05	0.12	1.20	0.15
*Per tweet*	–	–	1.31	0.20	0.04	0.08	0.83	0.11

Overall, as shown in the bottom section of Table 2, Supporter accounts tweeted much more often than other accounts, and used more hashtags, mentions, quotes, replies and URLs, but retweeted less often than both Opposers and Unaffiliated accounts. This suggests that Supporters were generating their own content (not just retweeting it), and attempting to engage with others through the use of platform features, implying a high degree of motivation on their part. These mere frequencies do not provide an understanding on who was being interacted with, the nature of those interactions or their content.

#### Interaction networks

Clustering and indexing are two main approaches to exploratory network analysis. Clustering revealed the polarised groups that form the basis of this study. Indexing includes centrality measures, *k*-core analysis and homophily metrics, which we employ in this section.

**Fig. 7:**
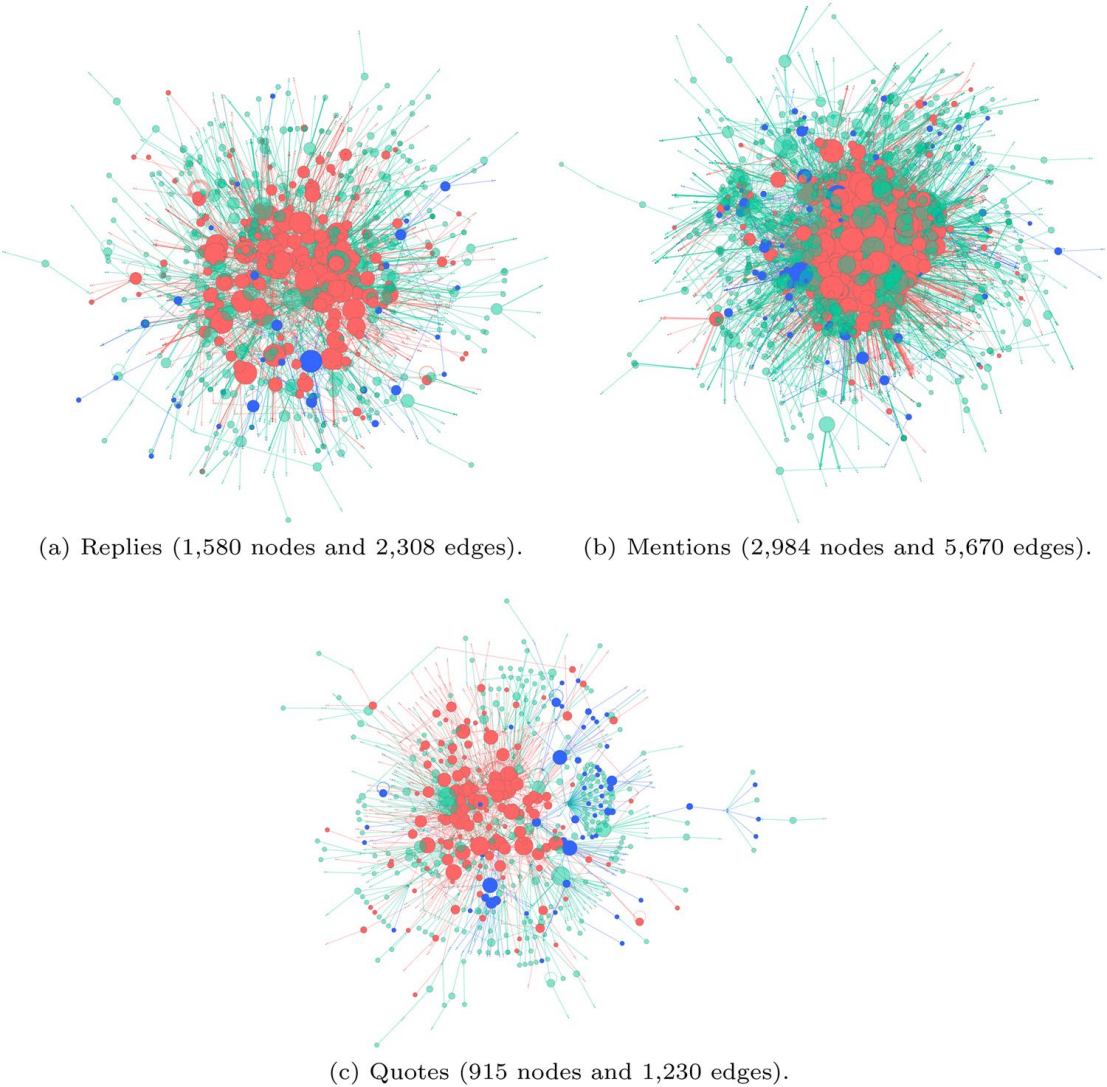
The largest connected components from directed, weighted networks built from the replies, mentions and quotes, linking from one account to another when it replied, mentioned or quoted the other. Edges are sized by weight, indicating the frequency of connections, and coloured by source node affiliation. Thicker edges have greater weight. Nodes are sized by outdegree (indicating the replies, mentions and quotes they used) and coloured by affiliation: red nodes are Supporters, blue are Opposers, and green are Unaffiliated

If Supporters employed a variety of interaction mechanisms, while Opposers relied primarily on retweeting, then Supporters should be deeply embedded in networks constructed from those other interaction mechanisms. For each of these, we inspect force-directed visualisations of the networks to see how clustered and deeply embedded the different groups’ members are, and then provide a degree of statistical rigour with various centrality and *k*-core measures partitioned by group and homophily measures of each groups’ insularity. We find Supporters are more embedded when we examine the largest components of networks constructed from replies (Fig. 7a), mentions (Fig. 7b) and quotes (Fig. 7c). These largest components include 77.4%, 92.0% and 72.2% of the reply, mention and quote networks’ nodes, respectively. Supporters had more connections and were clearly more active than Opposers using these interactions, engaging with each other and others in the network. They are particularly tightly and centrally clustered in the mention network, which is a reflection of their attempts to actively engage directly (rather than only indirectly, such as with hashtags). They are more diffusely located in the reply network, and the quote network, sharing similar network positions to Unaffiliated accounts. This is less to do with the amount of activity (i.e. the number of replies or tweets) and more to do with how they connect with others. The Opposer accounts that appear in the networks are not as centrally located nor as tightly clustered.

Centrality measures provide an indication of the importance of a node within a network, while the *k*-core of a node describes how deeply embedded it is within its network based on its connectivity^11^ (Newman 2010). Acting as a homophily measure, the Krackhardt E-I Index (Krackhardt and Stern 1988) is a simple ratio of edges internal to a community (i.e. between community members) and edges external to that community (i.e. edges which have only one endpoint within the community). In a graph, $G=(V,E)$, for a community *c* consisting of a nodeset $V' \subseteq V$ and for which the corresponding edgeset is $E' = \{ (v_i, v_j) \; |\; v_i \in V' \}$, its internal edges are $E'_{\mathrm{int}}(c) = \{ (v_i,v_j) \; |\; v_i, v_j \in V' \}$ while its external edges are $E'_{\mathrm{ext}}(c) = \{ (v_i,v_j) \; |\; v_i \in V', v_j \in V - V' \}$.^12^ Its E-I Index EIidx is given by

1 $$\begin{aligned} \text {EIidx}(c) = \frac{|E'_{\mathrm{ext}}(c) |- |E'_{\mathrm{int}}(c) |}{|E'_{\mathrm{ext}}(c) |+ |E'_{\mathrm{int}}(c) |} \end{aligned}$$

The E-I Index lies within $[-1,1]$ and low E-I Indices indicate highly homophilous networks in which nodes connect mostly with others in the same group. Our E-I Index implementation addresses the availability of edge weights^13^ by summing the weights of edges (rather than just their number). We refer to this measure as the *modified* E-I Index in the remainder of this work.

*Centrality* Though the location of Supporter and Opposer accounts in the networks in Fig. 7 gives the impression that Supporters are more central in each network, the statistics presented in Table 3 facilitate a more nuanced interpretation. Supporters had mostly higher betweenness and degree centrality scores, reflecting their more deeply embedded positions in the network, while Opposers were more efficiently connected to others (with higher Closeness centrality) and to more important nodes (eigenvector centrality) in most networks, despite their peripheral locations. The centrality scores suggest that the Opposers were less centrally located, but well connected, while Supporters were more centrally positioned (reflected in their relatively high betweenness scores).

**Table 3:** Mean centrality scores for Supporter and Opposer nodes in the largest components of the reply, mention and quote networks, omitting Unaffiliated node scores

Network	Group	Nodes	Centrality			
			Betweenness	Closeness	Degree	Eigenvector
Replies	Supporters	231 (14.6%)	0.000181	0.002871	0.004551	0.001307
	Opposers	82 (5.2%)	0.000019	0.003453	0.002757	0.001811
Mentions	Supporters	284 (9.6%)	0.000304	0.005525	0.004207	0.006575
	Opposers	140 (4.7%)	0.000018	0.005067	0.001997	0.006625
Quotes	Supporters	169 (18.5%)	0.000012	0.001876	0.006170	0.016033
	Opposers	80 (8.7%)	0.000005	0.003334	0.004171	0.007302

**Fig. 8:**
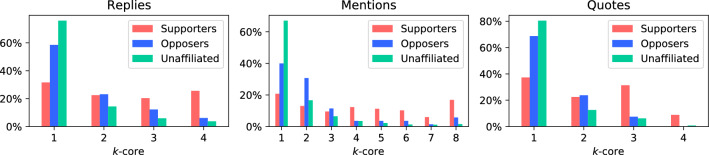
The distributions of *k*-core values for accounts in the reply, mention and quote networks. Nodes with higher *k*-core values are more deeply embedded in their network. The percentage refers to the proportion of each group’s accounts with a given *k*-core value

*k-core analysis* The question of how tightly clustered the nodes are can be addressed with *k*-core analysis. This analysis progressively breaks a network down to sets of nodes that have at least *k* neighbours, so nodes on the periphery are discarded first, while highly connected nodes form the ‘core’ of the network. Figure 8 shows the proportions of each groups’ members (of those present in each network) in each core. While the majority of Opposers and Unaffiliated are on the periphery of the networks, Supporters are relatively evenly spread throughout the networks’ cores. This implies some Supporters were more active than others in reaching out to other accounts, which is something not immediately visible in Table 2.

**Fig. 9:**
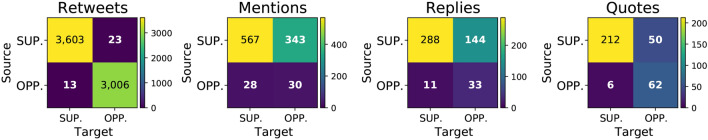
The mixing matrices of Supporter (SUP.) and Opposer (OPP.) interactions

*Homophily measures* The homophily measures introduced provide an indication of how insular the groups were with their interactions, and here we also apply them to the retweet network for comparison (Table 4). Within the retweet network, both communities were highly insular, retweeting in-group accounts almost exclusively, both when considering only the polarised groups and the broader network. Insularity amongst the other interactions distinguished the groups. Though preferring in-group connections, Supporters engaged more with Opposers than vice versa, when considering just the two polarised groups, but both connected with the broader network much more than in-group members, with Supporters leading the outreach in replies and quotes, while Opposers mentioned others more. Examining the mixing matrix of raw interaction counts in Fig. 9 emphasises the lower numbers of Opposer interactions and while the Opposer numbers were low, they very strongly preferred to reply and quote their own members. Other than when using mentions, Supporters clearly interacted with Opposers and Unaffiliated accounts more. Given Supporters opinions aligned with conservative politics (certainly with conservative news media, as we shall see later), this finding seems to go against other studies of political polarisation (Boutyline and Willer 2016).

**Table 4:** Homophily measures calculated with just Supporters and Opposers and then all nodes within interaction networks

Network	Polarised groups only					Broader network			
	Nodes		Edges	E-I Index		Nodes	Edges	E-I Index	
	Supporters	Opposers	Total	Supporters	Opposers	All	Total	Supporters	Opposers
Retweet	493	592	6645	− 0.98731	− 0.99139	12,076	21,526	− 0.70961	− 0.88997
Reply	247	105	476	− 0.33333	− 0.50000	2041	3031	0.62030	0.40541
Mention	288	149	968	− 0.24615	− 0.03448	3206	7523	0.69557	0.78723
Quote	190	104	330	− 0.61832	− 0.82353	1268	1542	0.45501	0.10791

Edge totals are the sums of the edge weights

#### The concentration of voices

The concentration of narrative from certain voices requires attention. Previous analysis (Weber et al. 2020) showed Supporters and Opposers were remarkably consistent in the phases in which they were most active, not in terms of how large a pool of accounts they retweeted from, but in how often they retweeted those accounts. Unaffiliated accounts retweeted accounts with greater frequency in all phases, implying that they often played a support role in disseminating content, becoming unwitting ‘participants’ in campaigns (Starbird et al. 2019).

Closer investigation (details in Supplementary Information, or SI) shows that Supporters were initially the most retweeted accounts, prior to the intervention, but after the intervention Opposers took their place. In Phase 3, especially, although Supporters posted nearly twice the number of tweets as Opposers, they contributed less than half the retweeted tweets. Additionally, after the intervention, Supporters made up nearly half the most retweeted accounts, but they were retweeted far less often than the Opposers. Supporters and Opposers clearly dominated the most retweeted accounts, however, thereby driving the major narratives of the discussion.

### Content dissemination

When contrasting the content of the two affiliated groups, we considered the hashtags and external URLs used. A hashtag can provide a proxy for a tweet’s topic, and an external URL can refer a tweet’s reader to further information relevant to the tweet, and therefore tweets that use the same URLs and hashtags can be considered related.

#### Hashtags

**Fig. 10:**
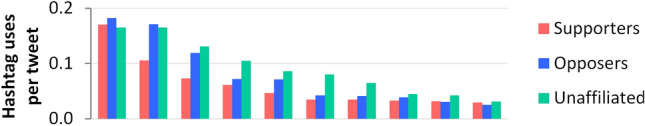
Hashtag uses per tweet for the ten most used hashtags for Supporters, Opposers and the Unaffiliated, omitting #ArsonEmergency. Opposers used hashtags more frequently than Supporters, but after the second hashtag, Unaffiliated accounts used more than either polarised group

Hashtag use was explored in our previous work through visualisations of hashtag networks (Weber et al. 2020). Our analysis found that Supporters used a wide variety of hashtags together, which we speculated was a way to inject the arson counter-narrative into different communities (Conover et al. 2011). Opposers were more focused on #AustraliaFires, #ClimateEmergency and a prominent media owner. These findings have now been confirmed statistically. Even though Supporters used approximately the same number of hashtags per tweet as Opposers (2.92 compared with 2.89), they used 40.9 hashtags per account, including 1.30 unique hashtags per account. In contrast, Opposers only used 17.5 hashtags per account, including 0.36 unique ones. This indicates the pool of hashtags used by the Opposers was much smaller than that of Supporters. The distribution of hashtag uses for the ten most frequently used by each group (which overlap but are not identical), omitting the ever-present #ArsonEmergency, is shown in Fig. 10. It indicates that Opposers focused slightly more strongly on a small set of hashtags, while Supporters spread their use of hashtags over a broader range (and thus their use of even their most frequently used hashtags is less than for Opposers). Unaffiliated accounts used their frequently used hashtags more often than both groups by the 4th hashtag, possibly due to the much greater number of accounts being active but less focused in their hashtag use. A second hashtag appeared in fewer than 20% of each groups’ tweets.

Further analysis of hashtag use, including as part of inauthentic behaviour, is addressed in Sect. 4.

**Fig. 11:**
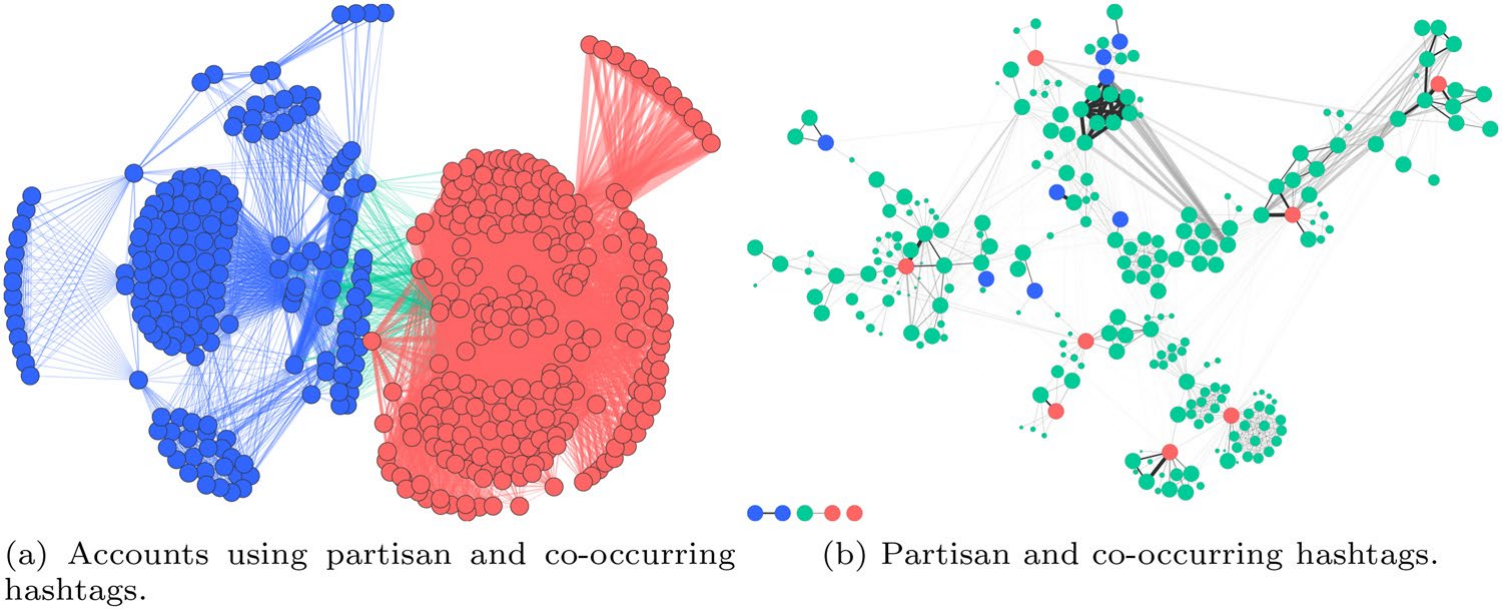
Two networks built from the tweets containing ‘partisan’ hashtags (minus the ten most common hashtags)

A statistical examination of how Supporters and Opposers used hashtags also revealed significant levels of homophily when considering only Supporters and Opposers, but less so when the hashtags use of Unaffiliated accounts was included. We created a network of accounts linked when they used the same hashtag (Fig. 11a, including only Supporter and Opposer accounts) and a network of hashtags used by the same account, though not necessarily in the same tweet (Fig. 11b). Rather than using all hashtags, we focused on those most used by each polarised group, starting with the ten most used by each *and* unique to each. Considering only those tweets with these 20 hashtags,^14^ and the other hashtags that occurred in the same tweets, minus the 10 most common hashtags overall (totalling 245 hashtags), we considered how much the groups’ discussions overlapped by topic. Network construction is detailed in SI.

Figure 11a’s nodes are accounts, linked when they mention the same hashtag. Red nodes are Supporters, while blue ones are Opposers, and edges are coloured according to their endvertices (green edges span the groups). Edge width represents edge weight, and isolates have been removed. Figure 11b’s nodes are hashtags, linked when they are used by the same account. Blue and red nodes represent the Opposer- and Supporter-specific partisan hashtags, respectively. Green nodes are co-occurring hashtags. Nodes are laid out with the backbone algorithm (Serrano et al 2009; Nocaj et al. 2014), and edges are shaded by backbone strength. The small components were joined only via the removed common hashtags.

Though some polarisation should be expected given the partisan hashtags provide a natural axis of polarisation, in the account network it is notable quite how little overlap there is in the use of the co-occurring hashtags. The clusters apparent in the account network (Fig. 11a) are caused by the fact that partisan hashtags are rarely mentioned by the same account (Fig. 11b). Instead they are clearly used with a variety of distinct hashtags, implying that although Supporters and Opposers were polarised in their hashtag use, they also had distinct sub-communities within their discussions (using hashtags as a proxy for discussion topic).

E-I Indices based on the full account network of 12, 867 nodes and 424, 389 edges for Supporters and Opposers were 0.147 and 0.717, respectively. Both groups used non-partisan hashtags, but Opposers used more popular ones more frequently, hence the higher E-I Index. Considering the subnetwork of only the 114, 797 edges between Supporters and Opposers causes the E-I Indices to fall to $-0.991$ and $-0.883$, respectively, indicating very little overlap in partisan hashtag use, and thus clear evidence of polarisation. These results are clearly evident in a visualisation of the network (Fig. 11a).

Considering the co-occurring hashtags again, we can see the clusters in the account network (Fig. 11a) are caused by the fact that the accounts rarely used multiple partisan hashtags together (otherwise there would be clusters of partisan hashtags); instead, whenever a tweet included a partisan hashtag, they also included one or a few of a variety of non-partisan hashtags, which are represented by clusters of green nodes in Fig. 11b.

#### External URLs

Our previous analysis (Weber et al. 2020) categorised *external* URLs (URLs in tweets that refer outside of twitter.com) as arson NARRATIVE aligned, CONSPIRACY content, DEBUNKING content and OTHER. The categorisation was based on the perceived *intent* of the use of the article, rather than purely on their factual content. Examining the frequency of the ten most shared URLs by each group in each phase, we found Unaffiliated accounts shared mostly NARRATIVE URLs in the first phase, but mostly DEBUNKING articles in the final phase (p.11, Weber et al. 2020).

This suggests that the new Unaffiliated accounts arriving in the final phase (discussed in Sect. 3.1 above) held different opinions on the arson narrative from the Unaffiliated accounts active early in the discussion. In fact, it is possible they acted as bridges bringing in new Opposer accounts—411 of the 585, or approximately 70% of Opposer accounts active in Phase 3, were not active in earlier Phases.

**Fig. 12:**
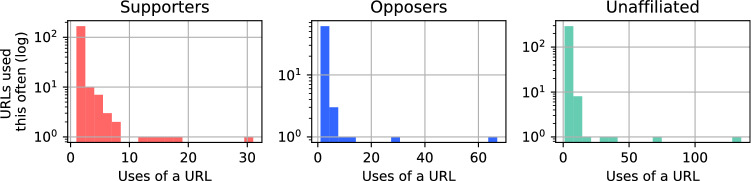
Distributions of URL use by Supporters, Opposers and Unaffiliated accounts

For each unique URL used, Supporters, Opposers and Unaffiliated groups had a mean rate of URL use of 2.0, 3.1 and 2.7, respectively, meaning Opposers were more focused in their URL use. This is evident in the distributions of URL uses in Fig. 12, which shows Supporters used more URLs more often that Opposers, and Opposers focused many of their uses on a small number of URLs.

### Coordinated dissemination

To investigate whether coordinated dissemination of content was occurring, we performed co-retweet, co-hashtag and co-URL analyses (Weber and Neumann 2021), searching for sub-communities of accounts that retweeted the same tweets, and shared the same hashtags, URLs and URL domains within the same timeframes (denoted by $\gamma$). The analyses result in weighted networks consisting of the sub-communities as disconnected components of accounts, the edge weights of which indicate the frequency of co-linking or co-mentioning of a hashtag. Further, to examine how the sub-communities relate to one another, we can then re-introduce the URLs and domains as explicit ‘reason’ nodes into these networks, making them bigraphs in which communities are joined according to these ‘reason’ nodes (Weber and Neumann 2021).

#### Co-retweet analysis

**Fig. 13:**
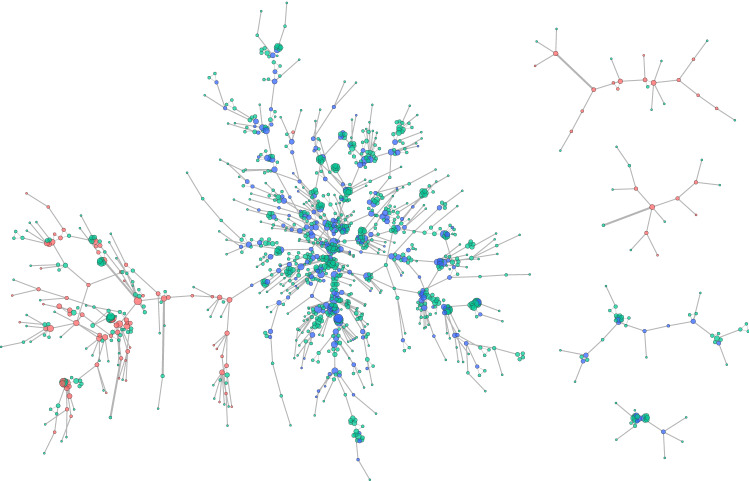
The five largest connected components of the co-retweet coordination network ($\gamma =1$ min), limited to only Supporter and Opposer accounts, which are sized by indegree. Red nodes are Supporters, blue are Opposers, and edges are sized by frequency of co-retweeting

The largest components of the co-retweet network ($\gamma =1$ min) shown in Fig. 13 show that the polarisation observed in the retweet network (in Fig. 3) is still evident, as expected, but what is particularly notable is the absence of tight cliques amongst the Supporter nodes, which, as promoters of the arson narrative, were originally thought to include a large proportion of bots (Stilgherrian 2020; Graham and Keller 2020). Cliques would indicate accounts all retweeting the same tweets within the same timeframe, a signal associated with automation, but also with high popularity (i.e. increasing the number of interested accounts increases the chance that they co-retweet accidentally). Cliques are visible amongst the 103 Opposers and many of the 966 Unaffiliated accounts (and could also be due to simple popularity and coincidence), but rare amongst the 233 Supporters. Instead their connection patterns imply real people seeing and retweeting each others retweets. For example, account A sees a tweet and retweets it, which is then seen by account B (within 1 min), and then account C sees that and retweets it as well, but longer than 1 min after A. A 1-min window is quite large for the purposes of identifying botnets, so this would indicate a lack of evidence of retweeting bots amongst the Supporters.

A further item to note is the degree of support offered by the Unaffiliated accounts, which co-retweet with Opposer accounts far more frequently than Supporter accounts in the coordination networks presented in Fig. 13. This observation raises the question of whether some of the Unaffiliated accounts may, in fact, be Opposers, but were simply not captured in the application of conductance cutting community detection to the retweet network, and they may have been captured with modification of the detection parameters.

#### Co-hashtag analysis

**Fig. 14:**
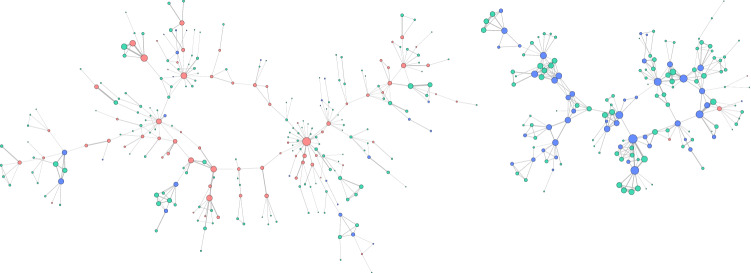
The two largest connected components of the co-hashtag coordination network ($\gamma =1$ min, excluding #ArsonEmergency), with nodes sized by the number of tweets they posted in the discussion. Red nodes are Supporters, blue are Opposers, green are Unaffiliated, and edge widths are sized by the frequency of co-hashtag activity

As using a hashtag in a tweet can increase its reach to observers of the hashtag as well as one’s followers, coordinated promotion of a hashtag is a mechanism to disseminate one’s message, thereby introducing a change of narrative (Conover et al. 2011; Varol et al. 2017), as well as pollute a discussion space (Woolley 2016; Nasim et al. 2018). Given how frequently hashtags are used, we chose a tight timeframe of 1 min and excluded #ArsonEmergency from our co-hashtag analysis. The two largest components discovered highlight the polarisation between the Supporter and Opposer communities (Fig. 14). The ring formation amongst the Supporters and small node sizes indicate less activity including a wider variety of hashtags. Opposers are more active and focused in the hashtags they used. These findings emphasise the findings in Sect. 3.3.1 but also highlight the support of Unaffiliated accounts, the most active of which appear to support the Opposers.

#### Co-URL and co-domain analysis

**Fig. 15:**
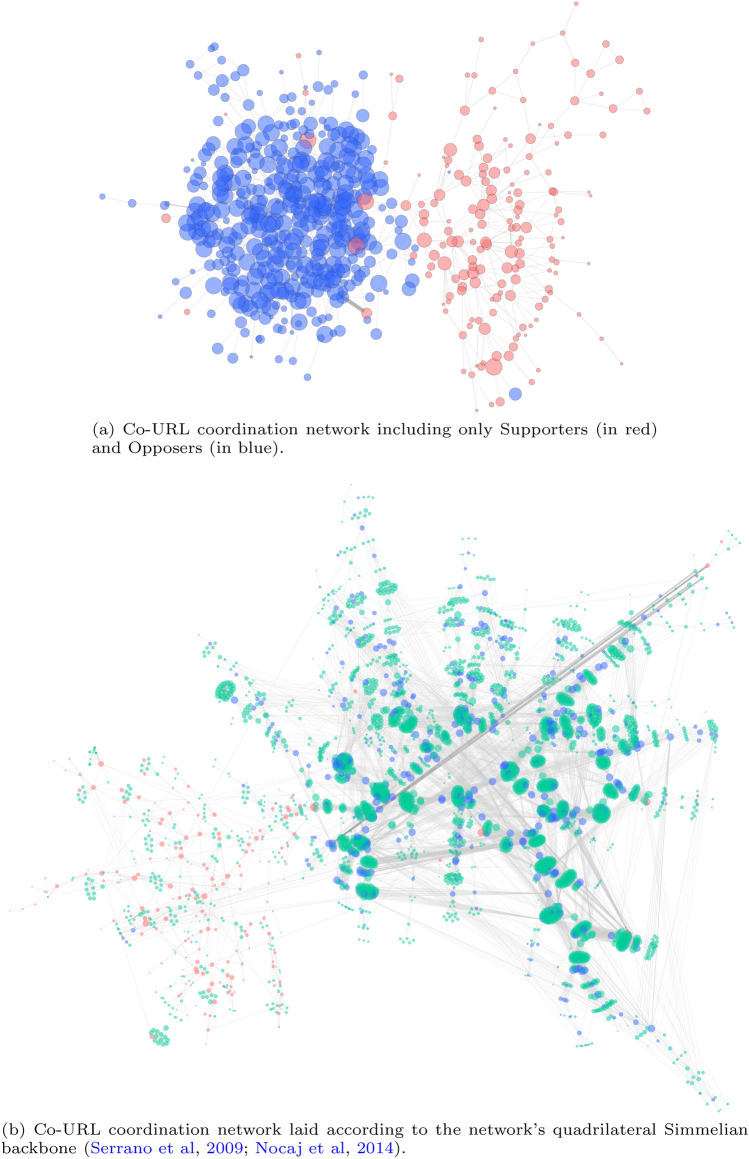
The coordination networks resulting from co-URL analysis ($\gamma =10$ min), with nodes sized by indegree. Red circular nodes are Supporters, blue are Opposers, and the green remainder are Unaffiliated accounts. Edge width and darkness indicates frequency of co-linking

For human users, grassroots-style coordinated co-linking should be visible in ‘human’ timeframes, such as within 10 min, allowing time for users to see each others’ tweets. The polarisation evident in the retweet network is also evident in the co-linking networks ($\gamma =10$ min) shown in Fig. 15, especially considering only the Supporter and Opposer networks (Fig. 15a). When we examine the co-linking in context in Fig. 15b, along with the contributions of Unaffiliated accounts, we can see that, again, Unaffiliated accounts co-acted with Opposer accounts far more often than Supporters, which appear relatively isolated, compared with the concentrated co-linking in the Opposer/Unaffiliated clusters on the right. Here, cliques represent groups of accounts sharing the same URLs, but it is unclear whether each clique represents a different URL or simply a different time window. To consider that, we need to introduce ‘reason’ nodes, representing the shared URLs, to create account/URL bigraphs.

**Fig. 16:**
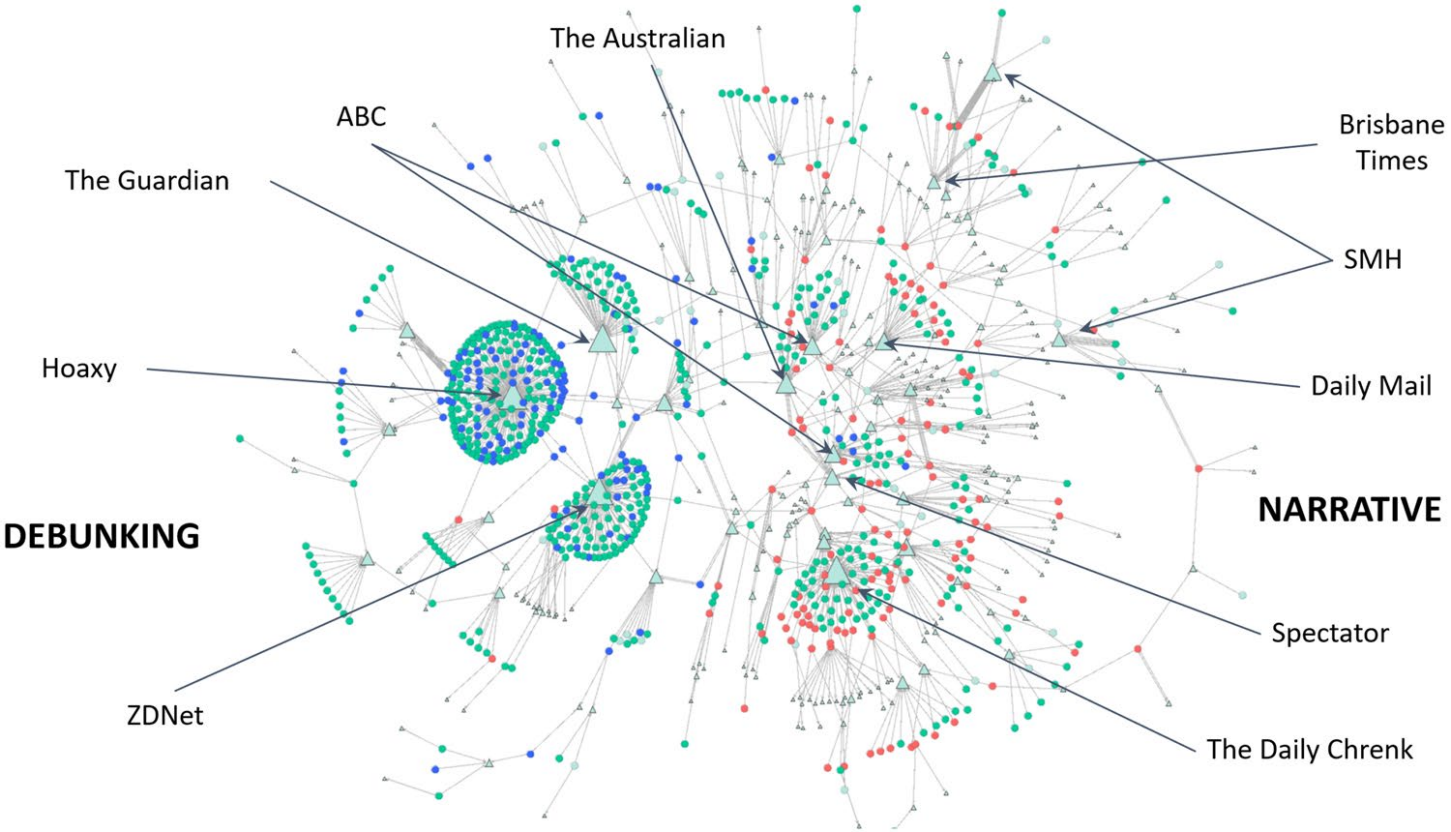
The account/URL bigraph resulting from co-URL analysis ($\gamma =10$ s), annotated with the websites hosting highly shared articles. Pale green triangular nodes are the URLs, sized by indegree. Red circular nodes are Supporters, blue are Opposers, and the green remainder are Unaffiliated accounts

**Fig. 17:**
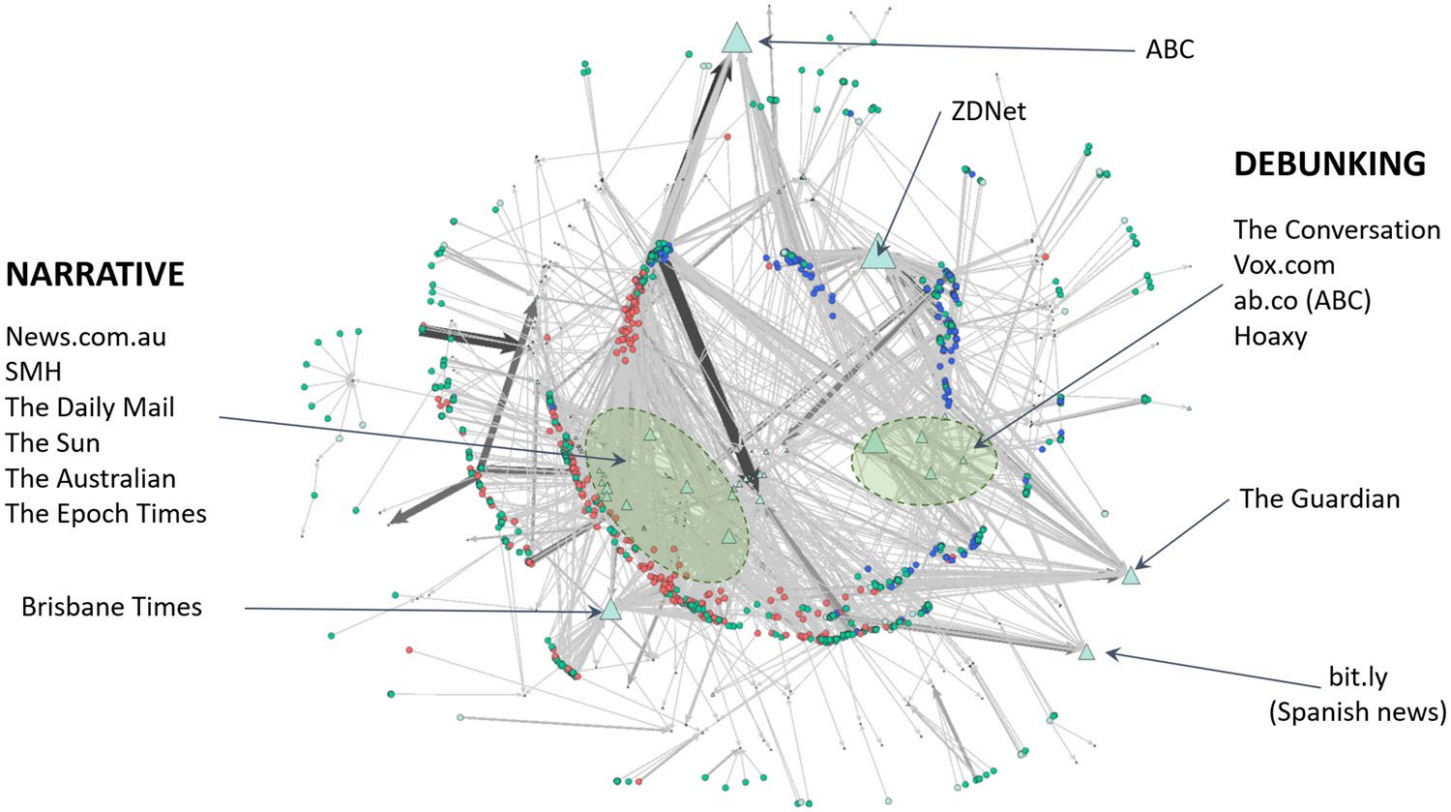
The account/domain bigraph resulting from co-domain analysis ($\gamma =10$ s), annotated with the websites hosting highly shared articles. Pale green triangular nodes are the URL domains, sized by indegree. Red circular nodes are Supporters, blue are Opposers, and the green remainder are Unaffiliated accounts. Two zones of contrasting highly linked to domains are highlighted, one primarily used to support the arson narrative, and one used primarily to debunk it

Figure 16 shows the resulting account/URL bigraph, which includes annotations indicating the websites hosting the most shared articles (referred to by the URLs). As with previous studies of rumour-based discussions, the most shared URLs often refer to MSM content and official reports (Hunt et al 2020). As expected, there is clear polarisation around the URLs, but it is immediately also clear how focused the Opposer accounts were on a small number of URLs, similar to their use of hashtags. The blue Opposer nodes link mostly to three URLs: the original ZDNet article (Stilgherrian 2020), the Hoaxy website (Shao et al 2018) and an article on The Guardian relating to online misinformation during the bushfires,^15^ which suggests the intervention was successful in drawing attention to the discussion. The Supporter community’s use of URLs is more dispersed, and includes MSM sites with the addition of a large cluster of Supporters and Unaffiliated accounts around an article on The Daily Chrenk, the website of an Australian blogger promoting the arson narrative. It is notable that two Australian Broadcasting Corporation (ABC) articles are so centrally located amongst the Supporters, as these were classified as DEBUNKING articles. When we consider the co-domain bigraph (Fig. 17), however, it is clear that the ABC domain binds the polarised Supporter and Opposer communities together, along with, interestingly, The Guardian and the URL shortener bit.ly. One bit.ly link appeared much more frequently than others, and it resolved to a Spanish news article on online bushfire misinformation.^16^ Highlighted in the co-domain bigraph are two zones of domains that appear mostly linked to one or the other of the Supporter and Opposer nodes, which are, again, appear polarised in the network. The domains in these zones appear aligned again with Opposers referring to domains hosting DEBUNKING URLs and Supporters referring to domains hosting NARRATIVE URLs. A few domains are referred to very frequently by individual nodes (visible as dark, large edges), and these are often social media sites, such as YouTube, Instagram and Facebook.

The analyses of a variety of co-activities here emphasise the polarisation observed in the retweet network permeates the groups’ collaborative efforts. Evidence indicates that Opposers, much more so than Supporters, engaged in coordinated action; however, given the significant contribution of Unaffiliated accounts, it is unclear whether this is deliberate or merely a reflection of high popularity (especially given the considerably greater number of Unaffiliated accounts active in the discussion).

## Inauthentic behaviour analysis

Coined by Facebook (Gleicher 2018), the term ‘inauthentic behaviour’ covers a range of activities that vary by motivation as well as the actions involved, but lacks a clear universal definition (Douek 2020). In essence, any interaction that is not in good faith could be regarded as inauthentic, rather than simply denouncing the use of automation or foul language. Examples include manipulating popular opinion during elections (Bessi and Ferrara 2016), ideological automated editing and vandalism of Wikipedia (Tsvetkova et al. 2017), as well as using sockpuppets to troll and bully (Kumar et al. 2017). The inauthentic behaviour we seek relates to deliberate text patterns in tweets. An expanded examination of the contribution of bots and the most bot-like accounts can be found in the SI. It is not included because it simply served to confirm the small influence of bots noted in our previous work (Weber et al. 2020), although several human-imitating social bots were clearly identified. Comparison with the dataset used for the ZDNet article was conducted, finding significant differences in the accounts considered. Details can be found in the SI.

**Table 5:** Frequency of inauthentic text patterns in the ArsonEmergency tweets (includes retweeted text)

	Phase 1		Phase 2		Phase 3		Overall	
	Count	% of All	Count	% of All	Count	% of All	Count	% of All
*Supporters*								
All	1573		121		5278		6972	
Hashtags	1	0.1	0	0.0	19	0.4	20	0.3
Hashtags + URL	160	10.2	7	5.8	502	9.5	669	9.6
Mentions + Hashtags	60	3.8	3	2.5	277	5.2	340	4.9
Mentions + Hashtags + URL	12	0.8	2	1.7	59	1.1	73	1.0
*Opposers*								
All	33		327		3227		3587	
Hashtags	0	0.0	0	0.0	0	0.0	0	0.0
Hashtags + URL	1	3.0	3	0.9	43	1.3	47	1.3
Mentions + Hashtags	0	0.0	0	0.0	0	0.0	0	0.0
Mentions + Hashtags + URL	0	0.0	0	0.0	5	0.2	5	0.1
*Unaffiliated*								
All	1961		759		14,267		16,987	
Hashtags	2	0.1	0	0.0	32	0.2	34	0.2
Hashtags + URL	181	9.2	14	1.8	434	3.0	629	3.7
Mentions + Hashtags	35	1.8	8	1.1	137	1.0	180	1.1
Mentions + Hashtags + URL	18	0.9	1	0.1	83	0.6	102	0.6

Aggressive language was observed in both Supporter and Observer content, but the hashtag and mention use provide the most insight into potential inauthentic behaviour (Gleicher 2018). Supporters used more hashtags and more mentions than Opposers in general (Table 2), and posted individual tweets with many more of each (50 tweets had at least 14 hashtags or 5 mentions), though a small proportion of Unaffiliated accounts used even more hashtags in their tweets (a maximum of 27). Overall, tweets that included more than 5 hashtags made up only $1.7\%$ of Opposer tweets, but $2.8\%$ of Supporter tweets and $2.1\%$ Unaffiliated tweets. Supporters posted tweets consisting of *only* hashtags, mentions and a URL in various combinations (i.e. eschewing actual content) far more frequently than Supporters or Unaffiliated, on a per-account basis, particularly in Phase 3 (see Table 5). Using hashtags and mentions in these numbers is a way to increase the reach of your message (though, ironically, it often leaves little space for the message itself), but can also be used to attack others or pollute hashtag-based discussion communities (Conover et al. 2011; Woolley 2016; Nasim et al. 2018).

In one notable instance, a Supporter account posted 26 highly repetitive tweets to an Opposer account within 9 min, including only the #ArsonEmergency hashtag in the majority of them. In six of the tweets, other accounts were mentioned, including prominent Opposer and Unaffiliated accounts, perhaps in the hope that they would engage by retweeting and thus draw in their own followers.

These findings make it clear that although Supporters were directly engaging with other accounts, their interactions did not always necessarily appear genuine. Supporters consistently tweeted just hashtags and URLs in around 10% of their tweets in the longer phases, and use of the other inauthentic text patterns grew between the first and last phases, possibly in response to the ZDNet article. Unaffiliated accounts in Phase 1 did the same, but use of that pattern dropped away in the later phases. Opposers rarely engaged in any of these text patterns.

Network analyses can reveal the existence of interactions between accounts, but not their nature. The sheer numbers of interactions prevent manual inspection; however, searching for text patterns such as those above, based on manual inspection of samples, can provide an indication of the authenticity or inauthenticity of the interactions, and are easy to detect. Further relatively simple analysis of use of specific hashtag sequences, e.g. ‘#ArsonEmergency #EcoTerrorism #ClimateChangeHoax’, in that order (*cf.*, Pacheco et al. 2021, case study 3), is another potentially simple yet informative analysis relying on sequence mining (Mooney and Roddick 2013).

Finally, as name switching had been observed in other discussions (Mariconti et al 2017; Ferrara 2017), we examined the accounts for such behaviour finding only 13 examples, including one Opposer and five Supporters. Some of the changes appeared to reflect a new ‘personality’ (*cf.*, Dawson and Innes 2019), but not in a particularly deceptive way—instead, the changes of name seemed whimsical.

## Discussion

Our discussion addresses the research questions we posed in Sect. 1.3. These extend the previous paper’s findings: that there were two clearly polarised communities in the discussion, which used different communication strategies, and the dominant narrative shifted from being arson-related to the official information from the first to the third phase.

**RQ1** *Behavioural differences over time and the impact of media coverage.*

Supporters were more active in Phases 1 and 3 and used more types of interaction than Opposers, especially replies and quotes, implying a significant degree of engagement, whether as trolls or as ‘sincere activists’ (Starbird and Wilson 2020). Unaffiliated accounts were consistently drawn in to the discussion in Phase 1, but most of these accounts left in the later phases and were replaced with many more Unaffiliated accounts who presumably joined based on reports in the MSM. Supporters’ use of interaction types remained steady from Phase 1 to 3. While behaviour remained relatively similar, activity grew for both groups after the story reached the MSM. The vast majority of accounts shared articles debunking the false narratives. The publication of the ZDNet article (Stilgherrian 2020) also affected activity, spurring Opposers and others to share the analysis it reported.

**RQ2** *Position of communities in the discussion network.*

Supporter efforts to engage with others in the discussion resulted in them being deeply embedded in the discussion’s reply, mention and quote networks and having correspondingly high centrality values. Our *k*-core analysis showed they were evenly distributed throughout the networks, from the periphery to the cores. Despite Opposers staying more on the periphery of the networks, they maintained high closeness and eigenvector centrality scores, meaning they stayed connected to more of the network than Supporters and certainly to more important nodes in the network. Correspondingly, this may imply that Supporters, though being highly connected, were not connecting as efficiently as Opposers, in order to spread their narrative. Both Opposer and Supporter groups were highly insular with respect to each other, across a variety of network analyses, but they connected strongly to the broader community according to E-I Indices.

**RQ3** *Content dissemination and coordinated activity.*

Analyses of hashtag and URL use revealed further evidence of the gap between Supporters and Opposers, not just in terms of connectivity, as discussed above, but also in terms of narrative. Supporters used a variety of hashtags to reach greater audiences, to disrupt existing communication channels or to otherwise harass. In doing so, they exhibited less evidence of coordination than Opposers, who were focused in both the hashtags and URLs they used, supported by, or in concert with, the much greater number of Unaffiliated accounts. Analysis of co-activities (namely co-retweeting, and co-URL and co-hashtag instances) suggested a lack of botnets in the discussion and that some Unaffiliated and Opposers were coordinating their URL sharing, appearing together in cliques that are often attributed to automation (e.g. Pacheco et al. 2020). The apparent coordination could, however, be attributed to high levels of popularity driven by increased activity in Phase 3 (i.e. coincidence due to high numbers of discussion participants), and the co-activities of Supporters indicated the presence of genuine human users more than any automated coordination. Further analysis using account/URL bigraphs showed that Opposers and Unaffiliated were focused on sharing a small set of URLs, compared with Supporters’ greater variety. These findings imply the Supporter community members, for all they attempted to engage with others via replies, mentions and hashtags, becoming deeply embedded in the interaction networks, remained relatively isolated from a narrative perspective.

**RQ4** *The role of bots, trolls and inauthentic behaviour.*

We found very few bots and their impact was limited: only 20 of 2512 accounts were classified as bots, while 96.6% were confidently classified as human (CAP $< 0.2$), though several were clearly social bots. In fact, following the ZDNet article, Graham and Keller argued that (non-automated) trolls are the more insidious element of this campaign, providing evidence that #ArsonEmergency was created specifically to counter #ClimateEmergency (Graham and Keller 2020) and may even have been part of a broader disinformation campaign involving elements of the political and media elite (Keller et al. 2020). Aggressive language was observed in both affiliated groups, but troll-like tweet text patterns including only hashtags, mentions and URLs were employed far more often by Supporters, especially in Phase 3. Distinguishing deliberate baiting from honest enthusiasm (even with swearing) is non-trivial (Starbird et al. 2019; Starbird and Wilson 2020), but identifying targeted tweets lacking content is a more tractable approach to detect inauthentic and potentially malicious behaviour.

It is worth elaborating on a number of further issues raised in this study.

### A disinformation campaign?

There is good reason to believe that #ArsonEmergency was deliberately created (Graham and Keller 2020), forming a ‘data deficit’ (Smith et al. 2020) for the sharing of misinformation regarding the arson narrative. This could form an isolated echo chamber for recruiting a new user base and radicalising it. Then, once established, it could link into the broader discussions by using a variety of hashtags in their tweets, which is what we observed. Radicalisation may not have been the ultimate goal of this particular community, but the technique could equally be used to garner support. Large isolated communities of accounts have been discovered by researchers before,^17^ and moderate levels of activity could remain undetected, particularly if participants avoided using other hashtags in their #ArsonEmergency tweets (which would link to other hashtag communities). #ArsonEmergency was discovered because participating accounts were known to Graham and Keller . This study provides confirmation of the presence of trolling, but no direct evidence of disinformation (*cf.*, Graham and Keller 2020; Keller et al. 2020).

### Strategies for countering misinformation

By regarding the publication of the ZDNet article as an intervention, we can evaluate its success in countering misinformation on #ArsonEmergency. Supporter numbers and activity rose dramatically after the story reached the MSM, drawing in many overseas contributors and shifting towards more inauthentic behaviour patterns. In contrast, the Opposer response was swift and simple, focusing on retweeting links to the ZDNet article and other fact-checks and official information, as it became available. Opposer activity was highest in Phase 2, but may have helped provide content for the incoming Unaffiliated accounts to share. In this way, the Unaffiliated accounts eventually shared DEBUNKING articles much more frequently than NARRATIVE aligned ones in the third phase. This occurred despite great increases in activity by Supporters, including relatively more uses of hashtags, mentions, replies, retweets and quotes than in Phase 1.

At a high level, this situation involved a number of events:

1. Researchers noticed a misinformation-based hashtag (that there was an #ArsonEmergency);
2. They then observed the high proportion of bots in the slowly growing surrounding discussion; and
3. They discussed these findings with a technology magazine.

The bot analyses were preliminary and did not stand further scrutiny (Graham and Keller 2020; Weber et al. 2020),^18^ but only days later the researchers clarified that much of the behaviour may have been due to (human) trolls (Graham and Keller 2020), and later presented evidence to suggest that the activity may have been coordinated with a broader disinformation campaign (Keller et al. 2020). The initial article, however, was enough to draw public attention to it, initially through ZDNet’s audience, spurring the Opposer community to form. By mid-morning after the news reached the breakfast MSM, it had had time to spread around the world as well as become known amongst the broader Australian online community. By the end of Phase 2, official announcements refuting the information were reported, and these became the focal points of the Opposer and Unaffiliated URL sharing.

In this way, a counter-misinformation strategy could involve:

1. Identify a misinformation echo chamber unified by criteria such as hashtag, a Telegram channel or Facebook Group or Groups;
2. Publish preliminary broad but newsworthy analyses of it via a minor online communication channel (e.g. a publisher with a national but small audience) in the afternoon, giving the channel’s audience time to discuss it on social media in the evening;
3. Ensure it receives enough attention to draw MSM coverage by the following morning;
4. Ensure experts and officials are available with statements ready to refute the misinformation, thus providing official records of information that can be reported by the MSM;
5. Focus any dissemination efforts on a small number of reputable MSM reports on the official statements—using MSM reports rather than official statements allows for interrogation by the press, building trust in the counter-narrative, and use of a limited number of articles helps focus the URL sharing behaviour (which may also affect platform trending algorithms);
6. Consolidate the analysis findings later in the discussion to provide nuance to the original results—this will ensure the initial results are validated, further building trust; and
7. Ensure the online discussion is monitored to evaluate the effectiveness of the counter-efforts.

There are limitations with this strategy and reasons why its success in this case study may be tightly associated with the specific context. By the time the broader Unaffiliated community entered the discussion, not only were there official statements and fact-checks to share, but they had also seen months of coverage of the bushfires on televisions around the world, so in a sense they were already primed to reject claims that the fires were not unusual. The ‘AustraliaFire’ discussion provides a strong example of this: the hashtag campaign was underpinned by mass retweeting of a few tweets that focused on the suffering of people and animals, thus establishing a moral imperative to respond to the crisis, strengthened with emotion. To be successful then, counter-efforts should either prime the Unaffiliated somehow (this is probably typically uncontrollable) or rely on some aspect of the Unaffiliated community’s mindset that acts as the priming mechanism, whether it be appealing to widespread ethical standards, rational thinking and/or the use of reputable sources.^19^

### Recommendations for future studies

The Supporter and Opposer communities were relatively easy to label, based on manual inspection of their most retweeted accounts. In studies of larger datasets, other methods may need to be considered, relying on automated analyses or other cues. Textual analyses, such as topic modelling of profile descriptions, could reveal a community’s major interests, but such descriptions are often very terse and sometimes do not align with account behaviour. In their study of the 2017 German election, Morstatter et al. (2018) identified several very large clusters of accounts using Louvain (Blondel et al 2008), but then determined the content of each major cluster using a hierarchical topic modelling technique applied to the hashtags they used. As discussed in Sect. 3.3, hashtags can be considered proxies for discussion themes. Furthermore, the predominant language used in the clusters also helped reveal distinct German-speaking alt-right and English-speaking alt-right clusters.

Our previous analysis (Weber et al. 2020) indicated that Supporters used original content and personal engagement more than Opposers and were active throughout the discussion. The analyses of the discussion’s interaction networks in this work suggest Supporters were deeply and widely embedded. Yet the analysis of inauthentic behaviour revealed that significant portions of these interactions with the broader community were not constructive, and some could be considered clear harassment.

These findings emphasise the value of mixed-method analyses, including using complementary methods, which take advantage of their relative strengths.

### Methodological contributions

Methodologically, the approach taken in this paper has taken advantage of recent advances in network science, bolstered them with established network, statistical and bot analyses, and proposed text patterns as a simple approach to identifying inauthentic behaviour. This final element helps illuminate the tone of interactions between the Supporter community and those outside it, on which network analyses shed little light. We observed that Supporters’ inauthentic behaviour seemed to mostly increase after the intervention, particularly the targeting of tweets with @mentions. It remains unclear whether this contributed to the Unaffiliated accounts’ shift away from the arson narrative.

The co-activity analyses used in this study further validate the utility of the approach, which has gained attention recently (Weber and Neumann 2021; Pacheco et al. 2021; Magelinski et al 2021). Similar recent work has applied co-URL and co-domain analysis to expose information polluters on the basis of the news they disseminate (and the sources from which it comes) (Truong et al. 2022). The inclusion of a temporal constraint aids in identifying concerted coordination over grassroots coordination, but improvements could be introduced to account for the scale of the discussion and co-activity coincidence rates.

Further research is required to examine the dynamic aspects of the social and interaction structures formed by groups involved in spreading misinformation to learn more about how to better address the challenge they pose to society. Future work will draw more on social network analysis based on interaction patterns and content (Bagrow et al. 2019) as well as developing a richer, more nuanced understanding of the Supporter community itself, including revisiting the polarised accounts over a longer time period and consideration of linguistic differences. A particular challenge is determining a social media user’s intent when they post or repost content, which could help distinguish between disinformation intended to deceive, and merely biased presentation of data or misinformation that aligns with the user’s worldview.

## Conclusion

The study of polarised groups, their structure and their behaviour, during times of crisis can provide insight into how misinformation can enter and be maintained in online discussions, as well as provide clues as to how it can be countered. The #ArsonEmergency activity on Twitter in early 2020 provides a unique microcosm to study the growth of a misinformation campaign before and after it was widely known, forming a natural experiment. Here, we have shown that polarised groups can communicate over social media in very different ways while discussing the same issue. In effect, these behaviours can be considered communication strategies, given they are used to promote a narrative and represent attempts to convince others to accept their ideas. Supporters of the arson narrative used direct engagement to reach individuals and hashtags to reach groups with a wide range of URLs to promote their message, while Opposers focused on using retweets and a select set of URLs to counter their message. Supporter activities resulted in them being deeply embedded and distributed in the interaction networks, yet Opposers maintained high centrality and were supported by and appeared to coordinate with active Unaffiliated accounts. The counteraction appears to have been successful, with the predominant class of articles shared being shifted from narrative aligned in Phase 1 to debunking articles in Phase 3. Graham and Keller’s efforts to draw attention to the #ArsonEmergency discussion (Stilgherrian 2020), and the subsequent associated MSM attention, is likely to have contributed to this effect, given the significant increase in discussion participants in Phase 3. This highlights the value in publicising research into misinformation promotion activities.

## Supplementary Information

Below is the link to the electronic supplementary material.

Supplementary file 1 (pdf 811 KB)

## Data Availability

The datasets collected and analysed during the current study (the identifiers of the tweets, as per Twitter’s terms and conditions) are available at https://github.com/weberdc/socmed_sna.

## Footnotes

1 https://www.abc.net.au/news/2020-02-19/australia-bushfires-how-heat-and-drought-created-a-tinderbox/11976134.

2 https://www.smh.com.au/national/green-ideas-must-take-blame-for-deaths-20090211-84mk.html.

3 https://www.theguardian.com/world/2013/oct/24/greg-hunt-wikipedia-climate-change-bushfires.

4 https://theconversation.com/au/who-we-are.

5 https://www.theguardian.com/us-news/2020/sep/16/oregon-fires-armed-civilian-roadblocks-police.

6 https://edition.cnn.com/2021/08/11/world/wildfires-climate-change-arson-explainer-intl/index.html.

7 https://github.com/twintproject/twint.

8 Australian Eastern Standard Time.

9 This collection occurs in the days prior to the passing of the Withdrawal Agreement Bill, on 22 January, 2020. Source: https://en.wikipedia.org/wiki/Timeline_of_Brexit. Accessed 2022-02-15.

10 The ‘lang’ field is automatically populated by Twitter based on language detection. If insufficient content is available (e.g. the tweet is empty, or only contains URLs or mentions, ‘und’ is used to mean ‘undefined’).

11 ‘A *k*-core is a maximal subset of vertices such that each is connected to at least *k* others in the subset’ (Newman 2010, p. 196).

12 NB, incoming directed edges are not included in $E'_{\mathrm{ext}}$. If the edges are directed, we count only interactions ‘reaching outwards’ from inside the community, and if the edges are undirected (e.g. when using the same hashtag), then incoming edges are not distinct from outgoing ones.

13 Edge weights are ignored in the implementation of the E-I Index in the version of NetworkX (Hagberg et al. 2008) that we used, version 2.5, which is why we implemented our own.

14 Supporters’ ten most frequently used unique hashtags were #ItsTheGreensFault (326), #Victoria (123), #GlobalWarming (107), #TheirABC (78), #ClimateCultist (66), #IndianOceanDipole (66), #Greens (62), #ecoterrorism (54), #Melbourne (53) and #NotMyABC (52), while those of the Opposers were #BlackSummer (74), #FossilFools (34), #KoalasNotCoal (29), #ArsonHoax (28), #ArsonMyArse (23), #DontGetDerailed (23), #Smoko (20), #bots (19), #ArseholeEmergency (17) and #FossilFuel (14).

15 https://www.theguardian.com/australia-news/2020/jan/08/twitter-bots-trolls-australian-bushfires-social-media-disinformation-campaign-false-claims

16 https://www.muyinteresante.es/naturaleza/articulo/actualidad-las-fake-news-de-los-incendios-de-australia

17 E.g. a network of 350,000 bots swapping Star Wars quotes was found in 2017 (Echeverria and Zhou 2017).

18 In fact, the ZDNet article *could* be argued to be misinformation itself, albeit intended to expose the arson-related misinformation.

19 E.g. the ABC was the most trusted media organisation in Australia in March 2020 (sec. ‘Content, services and communities’, ABC 2020).

## Code availability

The code used in this work is available at https://github.com/weberdc/socmed_sna.
