# Supplementary material for: Promoting and countering misinformation during Australia’s 2019–2020 bushfires: a case study of polarisation
Source: Soc Netw Anal Min. 2022 Jun 24;12(1):64. doi: 10.1007/s13278-022-00892-x (PMC9243896; doi:10.1007/s13278-022-00892-x)
Supplement: Supplementary file 1 — (pdf 811 KB) [file 13278_2022_892_MOESM1_ESM.pdf]

Promoting and countering misinformation during Australia's 2019-2020 bushfires: A case study of polarisation

Derek Weber[1,2*], Lucia Falzon, Lewis Mitchell and Mehwish Nasim

[1*] School of Computer Science, University of Adelaide, North Terrace, Adelaide, 5005, South Australia, Australia.
[2] Defence Science and Technology Group, West Terrace, Edinburgh, 5111, South Australia, Australia.

*Corresponding author(s). E-mail(s): derek.weber@{adelaide.edu.au,defence.gov.au};

Abstract

This file contains information supplementary to the main article. This includes analysis of the datasets, use of retweets and URLs, account locations, meta-discussion, further detail regarding bot-driven and other inauthentic behaviour, and hashtag use.

1 Dataset Analysis

In Phase 1, Supporters used `#ArsonEmergency` nearly fifty times more often than Opposers (2,086 to 43), which accords with Graham and Keller's findings that the false narratives were significantly more prevalent on that hashtag

compared with others in use at the time (Stilgherrian, 2020; Graham and Keller, 2020). This use is roughly proportional to the number of tweets posted by the two groups, however (Table 2 in the main paper). Overall in that Phase, Supporters used 22 times as many hashtags as Opposers. In Phase 2, during the Australian night, Opposers countered with three times as many tweets as Supporters, including fewer hashtags, more retweets, and half the number of replies, demonstrating different behaviour to Supporters, which actively used hashtags in conversations. Manual inspection and content analysis confirmed this to be the case. This is evidence that Supporters wanted to promote the hashtag as a way to promote the narrative. Interestingly, Supporters, having been relatively quiet in Phase 2, responded strongly, producing 64% more tweets in Phase 3 than Opposers. They used proportionately more of all interactions except retweeting, including many more replies, quotes, and tweets spreading the narrative with multiple hashtags, URLs and mentions. In short, Opposers tended to rely more on retweets, while Supporters engaged directly and were more active in the longer phases.

In the immediate aftermath of the publication of the ZDNet article, Opposers responded to growing Supporter activity on `#ArsonEmergency` with high numbers of retweets, whereas Supporters had been gradually promoting their narrative. It is possible that the Opposer community reacted strongly to the article due to ideological alignment with the ZDNet tech magazine or awareness of the researchers behind the analysis, who are well known in the Australian Twittersphere, with nearly 1,700 followers between them (at the time of the analysis), and in the Australian media, where they are often quoted on matters of social media use. Graham was mentioned 59 times by Opposers but only 11 times by Supporters, for example, mostly after the ZDNet article was published and then again around the time their Conversation article

appeared, on the 10th of January (Graham and Keller, 2020).[1] Supporters reacted strongly and consistently for several days into Phases 2 and 3, posting 64% more tweets in Phase 3 than Opposers.

2 Most Retweeted Accounts

Table 1 Contribution of the 41 accounts retweeted more than 100 times.

	Groups	Accounts	Retweets	RTs / account	% of top 41	% of all retweets
**Overall**	Supporter	17	5,487	322.8	35.7%	25.5%
	Opposer	20	8,833	441.6	57.5%	41.0%
	Unaffiliated	4	1,030	257.5	6.7%	4.8%
	Total		15,350			71.3%
**Phase 1**	Supporter	37	2,373	64.1	97.2%	90.7%
	Opposer	2	47	23.5	1.9%	1.8%
	Unaffiliated	2	21	10.5	0.9%	0.8%
	Total		2,441			93.3%
**Phase 2**	Supporter	16	192	12	19.7%	18.9%
	Opposer	21	772	36.8	79.3%	76.1%
	Unaffiliated	4	9	2.2	0.9%	0.9%
	Total		973			95.9%
**Phase 3**	Supporter	15	3,755	250.3	28.5%	21.0%
	Opposer	19	8,110	426.8	61.6%	45.3%
	Unaffiliated	7	1,303	186.1	9.9%	7.3%
	Total		13,168			73.6%

Of the 41 accounts retweeted more than 100 times, contributing 71.3% of the retweets, 17 were Supporters and 20 were Opposers (Table 1). The 20 Opposers were retweeted more overall and individually than Supporters, contributing the majority of the top retweeters tweets. This pattern was also apparent in the 25 accounts most retweeted by Unaffiliated accounts in Phase 3 (accounts retweeted at least 100 times): 8 were Supporters and 14 were Opposers.

[1] Graham and Keller themselves only posted 6 and 3 tweets in the dataset, and all were retweets after the ZDNet article appeared, and so their posts were not removed.

3 External URLs

URLs in tweets can be categorised as *internal* or *external*. Internal URLs refer to other tweets in retweets or quotes, while external URLs are often included to highlight something about their content, e.g., as a source to support a claim. By analysing the URLs, it is possible to gauge the intent of the tweet's author by considering the reputation of the source or the argument offered.

We categorised[2] the ten URLs used most each by the Supporters, Opposers, and Unaffiliated accounts across the three phases, and found a significant difference between the groups. URLs were assigned to one of these four categories:

NARRATIVE Articles used to emphasise the conspiracy narratives by prominently reporting arson figures and fuel load discussions.

CONSPIRACY Articles and web sites that take extreme positions on climate change (typically arguing against predominant scientific opinion).

DEBUNKING News articles providing authoritative information about the bushfires and related misinformation on social media.

OTHER Other web pages.

URLs posted by Opposers were concentrated in Phase 3 and were all in the DEBUNKING category, with nearly half attributed to Indiana University's Hoaxy service (Shao et al, 2016), and nearly a quarter referring to the original ZDNet article (Stilgherrian, 2020) (Figure 1a). In contrast, Supporters used many URLs in Phases 1 and 3, focusing mostly on articles emphasising the arson narrative, but with references to a number of climate change denial or right wing blogs and news sites (Figure 1b).

Figure 1c shows that the media coverage changed the content of the Unaffiliated discussion, from articles emphasising the arson narratives in Phase 1

[2] Categorisation was conducted by two authors and confirmed by the others.

to Opposer-aligned articles in Phase 3. Although the activity of Supporters in Phase 3 increased significantly, the Unaffiliated members appeared to refer to Opposer-aligned external URLs much more often. This suggests that the new Unaffiliated accounts arriving in the final phase (discussed in Section 3.1 in the main paper) held different opinions on the arson narrative from the Unaffiliated accounts active early in the discussion. In fact, it is possible they acted as bridges bringing in new Opposer accounts – 411 of the 585, or approximately 70% of Opposer accounts active in Phase 3 were were not active in earlier Phases.

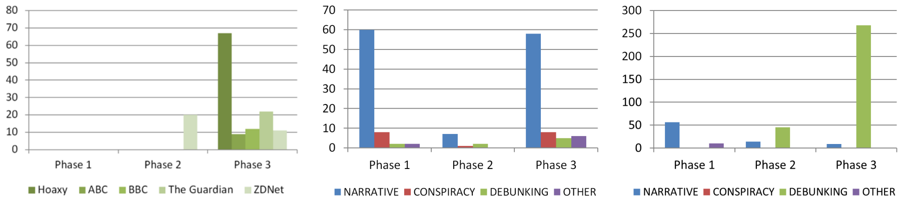

(a) Opposer URLs. (b) Supporter URLs. (c) Unaffiliated URLs.

Fig. 1 URLs used by Opposers, Supporters and Unaffiliated accounts (reproduced from Weber et al, 2020).

Supporters used many more URLs than Opposers overall (1,365 to 399) and nearly twice as many external URLs (390 to 212). Supporters seemed to use many different URLs in Phase 3 and overall, but focused much more on particular URLs in Phase 1. Of the total number of unique URLs used in Phase 3 and overall, 263 and 390, respectively, only 77 (29.3%) and 132 (33.8%) appeared in the top ten, implying a wide variety of URLs were used. In contrast, in Phase 1, 72 of 117 appeared in the top ten (61.5%), similar to Opposers' 141 of 212 (66.5%), implying a greater focus on specific sources of information. In brief, it appears Opposers overall and Supporters in Phase 1 were focused in their choice of sources, but by Phase 3, Supporters had expanded their range considerably. Ultimately, Supporters used 195 URLs

390 times (in total), Opposers used 68 URLs 212 times, and the Unaffiliated used 305 URLs 817 times, meaning a mean rate of URL use of 2.0, 3.1, and 2.7, respectively, meaning Opposers were more focused in their URL use. This is evident in the distributions of URL uses in Figure 12 (main paper), which Supporters use more URLs more often that Opposers, and Opposers focused many of their uses on a small number of URLs.

4 Location Analysis

In exploring the discussion of any contentious regional topic on social media, it is sensible to consider from where contributors come. People from different countries may bring different opinions to the table, and when such discussions may help shape public policy, there is the potential for malign foreign interference. The simplest approach is to consider the 'lang' field in the tweet metadata,[3] which is assigned by Twitter. Across every group and phase, roughly 99% of the tweets had a language code of 'en' (English) or 'und' (undefined). Manual inspection of the largest 'und' proportion (1,007 tweets by Supporters in Phase 3, 19.1% of those tweets) revealed the tweets' content comprised almost entirely of @mentions and hashtags.

To learn more, we examined the 'location' field in the 'user' objects in the tweets. This is a free text field users can populate as they wish and contains a great variety of information, not all of which is accurate, but the majority of populated fields are at least meaningful locations (88%). We manually coded the 'location' for each Supporter and Opposer account and then the 'location' values that appeared more than once for the Unaffiliated accounts (Table 2). The majority of contributors in each group is from Australia, but the Supporters and Unaffiliated accounts included more non-Australian but

[3] The 'language', 'utc_offset' and 'timezone' fields within the 'user' field of tweets have been deprecated: https://developer.twitter.com/en/docs/tweets/data-dictionary/overview/user-object.

Table 2 The self-reported locations of accounts, categorised by country by hand. Only non-empty locations were used, and only those used multiple times by Unaffiliated accounts were considered (i.e., unique Unaffiliated locations were ignored).

	**Opposer**		**Supporter**		**Unaffilated**	
Country	Counts	Proportion	Counts	Proportion	Counts	Proportion
Australia	393	88.7%	273	76.9%	3,642	72.0%
USA	4	0.9%	19	5.4%	586	11.6%
UK	4	0.9%	5	1.4%	287	5.7%
Canada	2	0.5%	7	2.0%	146	2.9%
NZ	2	0.5%	5	1.4%	51	1.0%
Miscellaneous	35	7.9%	41	11.5%	143	2.8%
Other	3	0.7%	5	1.4%	204	4.0%
Total	443	100.0%	355	100.0%	5,059	100.0%

English-speaking contributions than Opposers. The larger proportion of American and UK contributions in the Unaffiliated accounts may be due to an influx of highly-motivated users who joined the discussion after Graham's analysis (Stilgherrian, 2020) reached the MSM. It is thought that climate change is less settled in those countries.[4] This is borne out by the increased number of unique Unaffiliated accounts in Phase 3.

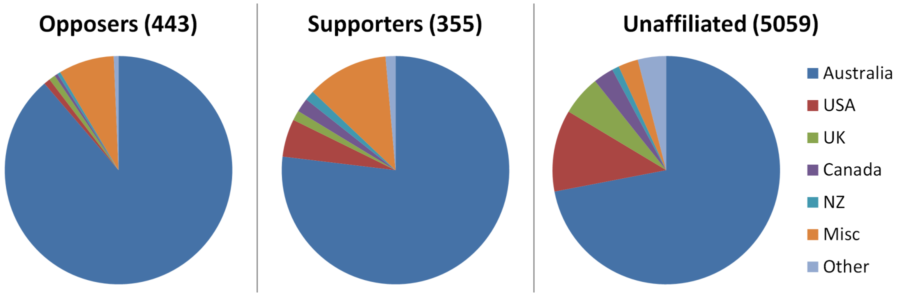

Fig. 2 The self-reported locations of Supporter, Opposer and Unaffiliated accounts. The number in brackets indicates how many accounts were evaluated. The Miscellaneous category was used for locations which described a physical location but were vague, e.g., Earth, whereas Other was used for whimsical entries, e.g., "Wherever your smartphone is." or "Spot X".

Given the global effect of climate change, any prominent contentious discussion of it is likely to draw in participants from other timezones. Although

[4]https://www.theguardian.com/environment/2019/may/07/us-hotbed-climate-change-denial-international-poll

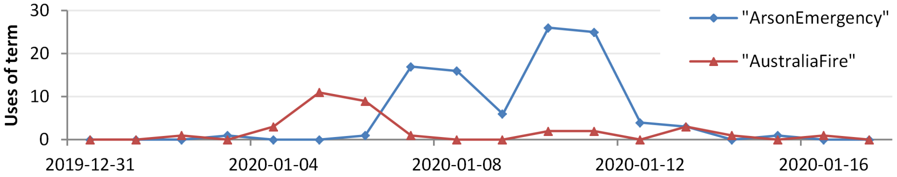

Fig. 3 Counts of tweets using the terms 'ArsonEmergency' and 'AustraliaFire' without a '#' symbol in meta-discussion regarding each term's use as a hashtag (counts outside were zero).

the activity patterns in Figure 1 (main paper) indicate the majority of activity aligns with Australian timezones, a deeper analysis of the self-reported account 'location' fields in tweets revealed that only 88% of active[5] participants were Australian (Figure 2). (Tweets can contain geolocation information but rarely do: only 127 tweets in the 'ArsonEmergency' dataset had any geolocation information, and 114 were posted in Australia.) Based on the self-reported location, more Supporters declared locations outside Australia (23%) than Opposers (11%), but the biggest proportion of non-Australian participants were Unaffiliated, perhaps drawn in by the international news. It is unclear whether the international accounts were drawn in to aid the Supporters or Opposers in Phase 3, but we know the articles the Unaffiliated shared changed to DEBUNKING in that Phase, and Unaffiliated accounts appeared to coordinate with Opposers.

4.1 Meta-discussion: Avoiding promotion of the hashtag

The terms 'ArsonEmergency' and 'AustraliaFire' (without '#') were used for the Twarc searches, rather than '#ArsonEmergency' or '#AustraliaFire', to capture tweets that did not include the hashtag symbol but were relevant to each discussion. This was done to capture discussions of the term, in which participants deliberately chose to avoid using the term in a way that would contribute to the hashtag discussion (i.e., by including the hashtag symbol).

[5]We considered all Supporters, Opposers, plus all Unaffiliated accounts that tweeted at least three times, and who populated the field.

We refer to this as meta-discussion, i.e., discussion *about* the discussion. We sought to understand how much of the discussion relating to `#ArsonEmergency` (and `#AustraliaFire`, for comparison) was, in fact, meta-discussion. Of the 27,546 tweets in the 'ArsonEmergency' dataset, only 100 did not use it with the '#' symbol (0.36%), and only 34 of the 111,966 'AustraliaFire' tweets did the same (0.03%), so it is clear that very little of the discussion was meta-discussion. That said, there were several days on which tens of tweets seemed to be involved in meta-discussion, as shown in Figure 3. These coincide with Phase 2, when the story reached the MSM, and then again a few days later, possibly as a secondary reaction to the story (commenting on the initial reaction to the story on the MSM), or as a reaction to the Conversation article (Graham and Keller, 2020). As such, we are confident many of these uses can be considered deliberate reactions to the discussions. Examination of these particular tweets confirms this; we present examples in Table 3.

Table 3 Examples of meta-discussion referring to the #ArsonEmergency hashtag without including it directly by removing or separating the leading '#' character.

Research from QUT shows that 'some kind of a disinformation campaign' is pushing the Twitter hashtag # ArsonEmergency. There is no arson emergency. https://t.co/⟨URL⟩
@⟨ACADEMIC⟩ @⟨JOURNALIST⟩ Venn Diagram of "ArsonEmergency" with "Qanon" and "Agenda21" conspiracies could be interesting ⟨UNIMPRESSED EMOJI⟩
suggest @AFP @NSWpolice ,@Victoriapolice as this misinformation is likely to cause panic & distress in Bushfire hit communties.<br>This link is US news but it contains saliant facts about arrests. https://t.co/⟨URL⟩<br>When retweeting, remove hashtag from 'arsonemergency' https://t.co/⟨URL⟩
@⟨JOURNALIST⟩ #!ArsonEmergency - a notag.

5 Exploration of Inauthentic Behaviour

We present details of bot analyses and comparisons with other research, further inauthentic text patterns in tweets, and analyses of the behaviour of accounts changing their screen names during the collection period.

5.1 The Contributions of Bots

We consider the same 2,512 (19.5%) of the accounts in the dataset, which were labelled according to their Botometer Complete Automation Probability (CAP) scores as 'bots' (≥ 0.6), 'humans' (< 0.2) or 'undecided' (≥ 0.2 and < 0.6). In contrast, the analysis conducted for the ZDNet article (Stilgherrian, 2020) used the `tweetbotornot`[6] R library and found far more bots. Previously, we found the significant majority of accounts had human CAP scores (Weber et al, 2020). Building on these findings, our aim is to first consider whether the bot accounts found had an oversized contribution to the discussion, and whether this contribution was consistent through the phases.

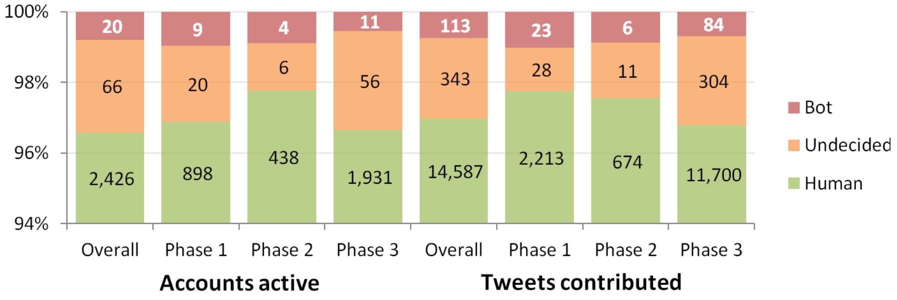

Fig. 4 The proportion of active human, bot and undecided accounts active and the proportion of tweets they contributed, both overall and in each phase. The data was sourced from Table 3 in (Weber et al, 2020).

Figure 4 shows that very little contribution was made by non-human accounts. The proportion of bot accounts and their tweet contribution consistently dropped over time, but the tweet contribution of undecided accounts grew consistently, but, even so, non-human accounts accounted for only up to approximately 3% of accounts and tweets. Statistical tests confirmed that the distributions of Botometer scores did not differ significantly between Phase 1 and overall, and between Opposers and Supporters, likely due to the dominance of the skew towards human accounts.

[6]https://github.com/mkearney/tweetbotornot

5.1.1 Discrepancies with the ZDNet results

The analysis in (Stilgherrian, 2020) suggested hundreds of bots were active on `#ArsonEmergency`, however the results presented here and in (Weber et al, 2020) indicate far fewer were present, and they were similarly distributed across the phased and within the polarised groups. The contrast between these results is likely to be due to a number of reasons, but the primary one is differences in our datasets. Graham and Keller used the collection tool Twint (which avoids using the Twitter API and instead uses the Twitter web user interface (UI) directly) to focus on results from Twitter's web UI when searching for `#ArsonEmergency`. Only 812 tweets appeared in both datasets, and even those were restricted to Phase 1. Of the 315 accounts in common, 100 were Supporters and 5 Opposers, implying that those Supporter accounts had already been flagged by misinformation researchers as having previously engaged in questionable behaviour. The size of our dataset and the greater number of accounts we tested is likely to have skewed our Botometer results towards typical users. There are also differences between the bot analysis tools. Botometer's CAP score is focused on non-hybrid, English accounts, whereas `tweetbotornot` may provide a more general score, taking into account troll-like behaviour. The content and behaviour analysis discussed above certainly indicates Supporters engaged more with replies and quotes, consistent with other observed trolling behaviour (Kumar et al, 2018; Mariconti et al, 2019) and "sincere activists" (Starbird and Wilson, 2020). Follow-up work by Graham and Keller's research group has focused on such "activists" and the contribution of trolls (Graham and Keller, 2020), finding that they appeared to coordinate their activities with prominent public figures and media outlets as part of a broader and longer-running disinformation campaign spanning the months surrounding the period we have focused on (Keller et al, 2020).

As our collection was performed via the Twitter Search API, rather than its Streaming API, and the first of those searches was on the 8th of January, it is possible, if not likely, that Twitter had already stripped some bots and their content from their data holdings. Furthermore, it is unclear whether Twitter results are 'cleaned' before being provided to those requesting them (Assenmacher et al, 2021).

Finally, it should be noted that at the time of writing the `tweetbotornot` library has been replaced with a new version in a completely separate library `tweetbotornot2`[7] in which the bot rating system has been changed and is now more conservative. In this way, the original findings in January 2020 may be been an artifact of the original implementation, however the polarised communities discovered since are certainly real and worthy of study.

5.1.2 The most bot-like accounts

Table 4 Supporter and Opposer accounts with a Botometer rating above 0.8. Counts of tweets, friends, and followers, and ages are as of the last tweet captured during the collection period in January, 2020.

	**Supporters**			**Opposers**	
	Bot 1	Bot 2[1]	Bot 3	Bot 1	Bot 2[2]
Contribution	5	9	59	4	4
Retweets	5	9	56	4	4
Age (in days)	1,081	680	1,087	1,424	925
Lifetime tweets	47,402	10,351	349,989	62,201	74
Tweets per day	43.85	15.22	321.98	43.68	0.08
Friends	17,590	13,226	25,457	633	392
Followers	16,507	13,072	24,873	497	55
Reputation	0.484	0.497	0.494	0.440	0.123

[1]This account was found to have been deleted when checked in October, 2020.

[2]This account was found to have been deleted when checked in December, 2020.

Deeper analysis of the most bot-like accounts (those with a CAP ≥ 0.8) revealed that the kinds of bot-like accounts present in each community differed

[7]https://github.com/mkearney/tweetbotornot2

significantly in a few primary respects (see Table 4). For convenience, we will refer to these accounts as "bots", but given all but Opposer bot 2 present as genuine human users, they may also qualify as "social bots" (Cresci, 2020) and therefore are likely to be tools for influence. The accounts were re-examined in late 2020, finding that two of the accounts had been suspended. Their profiles provide an indication of the accounts' interests and motivations. Supporter bots 1 and 2 clearly appeared to be fans of former US President Donald Trump, with many references to him in their profiles and tweets, while the third Supporter account claimed to be an indigenous Tasmanian grandmother, but whose tweets also supported Trump. The profile of Opposer bot 1 reflected a typical user with no indication of political leaning, but whose tweets were left-aligned. The other Opposer seemed to be a finance services marketing bot.

Together, the five accounts in Table 4 contributed 81 tweets over the 18 day collection period, 73 by the Supporters (including 59 from Bot 3) and 4 each from the Opposer bots. This suggests they had very limited opportunity to have an impact on the discussion. All accounts had been active for at least eighteen months, up to a maximum (at the time of the collection) of nearly four years. The variations in posting rates highlight the fact that Botometer's ensemble classifier will catch accounts that do not have high posting rates (e.g., Opposer bot 2 only posted approximately 25 tweets per year, but had been suspended by December, 2020). The *reputation* score is defined by

$$reputation = \frac{|followers|}{|friends| + |followers|}, \tag{1}$$

and is a measure considered to be desirable enough worth manipulating through follower fishing (Dawson and Innes, 2019), yet even the bots' reputation scores are not very different (other than Opposer bot 2, which seems to be

a rarely used account). In fact, the primary distinction between the Supporter and Opposer bots is the magnitude of their friend and follower counts.

Supporter bots had an average of 18.8k friends and 18.5k followers compared with Opposer bots' averages of 512.5 friends and 276 followers. By October, 2020, over nine months, the two remaining Supporter bots, bots 1 and 3, had increased their friend and follower counts significantly: bot 1 had 1.7k[8] more friends and 1.1k more followers, while bot 3 had 14.5k more friends and 13.4k more followers. Over the same period, bot 1 had posted another 36.3k tweets (a 77% increase at more than 130 tweets per day) and bot 3 had posted another 157.3k tweets (a 45% increase at nearly 600 tweets per day). Bots 1 and 3 had been created 6 days apart and, in January, 2020, both had been running for just over three years. In contrast, Opposer bot 1 had lost one follower and reduced the number of accounts it followed by 9, but added just over 10k tweets (approximately 37 tweets per day), while Opposer bot 2 had increased the accounts it followed by 148%, added one follower and posted only 25 tweets.

Figure 5 shows the activity patterns for the Supporter and Opposer bot accounts, and also for the 15 Unaffiliated accounts that had been suspended when the bot analysis was conducted (at the end of January 2020). The Opposer contribution is small and occurs in Phase 2 and the first day of Phase 3, clearly responding to the MSM news, while the Supporter bots are active in the lead up to Phase 2 and well into Phase 3, engaging in the ongoing discussion, though their activity patterns indicate that if they are bots tweeting frequently, then their tweets mostly avoided using `#ArsonEmergency` (and thus were not captured in our collection). The Unaffiliated accounts are also mostly active only on the day the story reached the MSM and the following day, and their contribution was limited to only 32 tweets.

[8] Count changes are in thousands, as the figures are obtained from the profile screenshots.

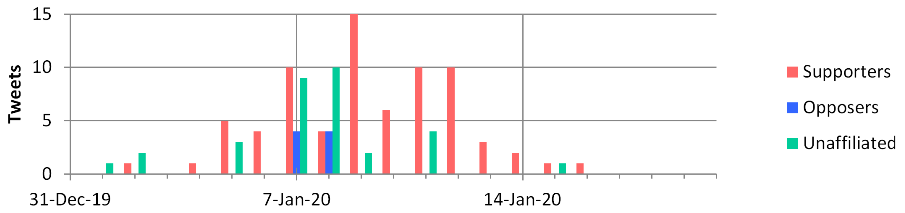

Fig. 5 Tweets per day by the three Supporter, two Opposer and fifteen bot accounts.

The five accounts contributed 81 tweets in 18 days, which were mostly retweets and the majority were from one Supporter, indicating their influence on the discussion was limited. Although Botometer's performance has started to diminish against newer bots (Feng et al, 2021), the use of automation by the bots is quite plausible, given the remarkably consistent and high posting rates of the accounts, their highly balanced follower and friend counts, and their activity between January and October, 2020 (Table 5). Opposer bot 2's tweeting motivations are unclear, but it may have been a bot account left dormant for later commercial use (e.g., for narrative switching, Dawson and Innes, 2019).

Table 5 Changes in bot accounts between January and October 2020. Details for Supporter bot 2 are missing as it had been suspended by October.

Account	Friends	Followers	Tweets	Tweets / day
Supporter bot 1	1.7k ↑	1.1k ↑	36.3k	130
Supporter bot 2	14.5k ↑	13.4k ↑	157.3k	≈ 600
Opposer bot 1	9 ↓	1 ↓	10k	37
Opposer bot 2	581 ↑	1 ↑	25	< 1

It is not clear why these accounts are so different. It is possible these accounts are, in fact, merely highly motivated people, who spend a significant amount of time curating their Twitter feeds to include material they prefer and then retweet almost everything they see to simply promote their preferred narrative. This accords with recent observations that Twitter increasingly consists of retweets of official sources and celebrities and tweets with URLs, and

rather than being a town square of public discussion, it should be treated as an "attention signal", which highlights the "stories, users and websites resonating" at a given time (Leetaru, 2019). These accounts appear driven to amplify that "attention signal" for ideological reasons, for the most part. What also stands out is that the Supporter bots differ distinctly from the rest of the Supporter community who relied much less on retweets than the Opposer community.

5.2 Non-genuine Patterns in Tweet Text

Aggressive and profane language was observed in content posted by both Supporters and Opposers, but our observations includes behaviour that could be regarded as inauthentic (Weedon et al, 2017), including trolling. We examined the frequency of hashtags and mentions appearing in tweets by Supporters, Opposers and the remainder of accounts, as well as identifying inflammatory behaviour through manual inspection.

The 288 Supporters and 149 Opposers in the mention network connected to Opposers and Supporters, respectively, slightly more than they mentioned themselves, with 710 edges (E-I Index of -0.14). When Unaffiliated accounts are considered (resulting in a mention network of 3,206 nodes and 5,825 edges, a subset of the one shown in Figure 7b (main paper) which omits Unaffiliated—Unaffiliated edges), the combined E-I Index for Supporters and Opposers rises to 0.7, suggesting a clear preference to mention Unaffiliated accounts.

An analysis of contemporaneous co-mentions also reveals that Supporter accounts mentioned the same accounts in quick succession much more frequently than Opposers, but that one prominent Opposer account was mentioned by many other accounts (Figure 6). It is clear the highly mentioned Opposer is a target for accounts, with many pairs of co-mentioners mentioning

only the Opposer. A second (Unaffiliated) account is also highly mentioned, lying just below the Opposer account, though it appears mentioned more often by Supporter accounts, while the Opposer is more often mentioned by Unaffiliated accounts. The Opposer account is a prominent left-wing online personality mentioned more than 2400 times in the dataset, while the Unaffiliated account had been suspended by the end of January 2020, just after the collection period, and was mentioned over 350 times in the dataset. The largest Unaffiliated mentioning account (circular green node, on the right of the large connected component) appears to support the arson narrative and also promotes a number of QAnon-related hashtags (The Soufan Center, 2021).

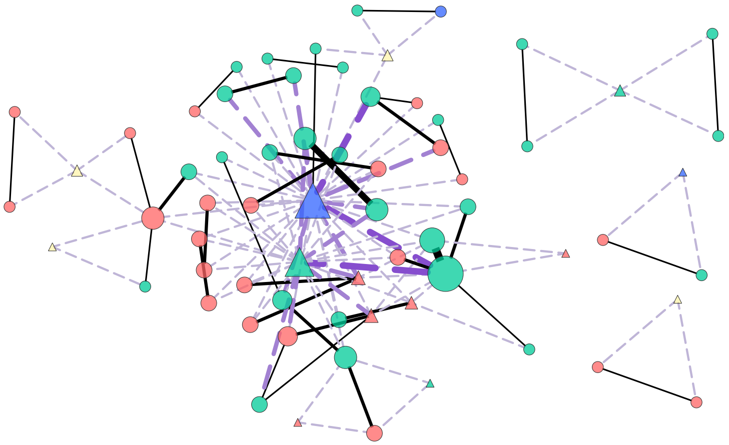

Fig. 6 The account/mention bigraph resulting from a co-mention analysis, connecting accounts with black edges when they mentioned the same account within 60 seconds. Purple edges connect accounts with the accounts they mention, which are shown as triangles. Node colour indicates affiliation: red nodes are Supporters; blue nodes are Opposers; green nodes are Unaffiliated accounts; and yellow nodes are accounts that were mentioned but did not post a tweet in the dataset. Node size indicates the number of tweets they contributed to the corpus or, for mentioned accounts, their degree (reflecting the number of times they were mentioned).

Tweets that include many hashtags or mentions can stand out in a timeline, because the vast majority of tweets include very few, if any. By including many hashtags, a tweet may be seen by anyone searching by those hashtags, thereby increasing its potential audience. Including many mentions may be

a way to draw other participants into an ongoing conversation or at least inform them of an opinion or other information. Figure 7 shows that all groups trended similarly, and that Supporters posted more tweets with many hashtags than Opposers did (although they tweeted nearly twice as often). Unaffiliated accounts used the most hashtags in tweets, with more than 100 Unaffiliated tweets including 19 or more hashtags. Given the great numbers of Unaffiliated accounts and tweets, these can be regarded as outliers (making up less than 1% of their contribution).

Supporters used many more mentions than Opposers more often (Figure 8). Opposers only used a maximum of 5 mentions on fewer than 10 occasions, while Supporters did the same more than 50 times. In fact, Supporters used more than 5 mentions in 369 tweets. In a few tweets, 45 or more mentions appear, however analysis of this phenomenon has revealed that Twitter accumulates mentions from tweets that have been replied to. One reply tweet including 50 mentions was a simple reply into a reply chain that stretched back to 2018. Many replies in the chain had mentioned one or two other accounts, and they were then incorporated as implicit mentions in any replies to them. Unfortunately, from the point of view of the data provided by the Twitter API, it is unclear whether mentions in a reply are manually added by the respondent or included implicitly, as they simply appear at the start of the tweet text.

Although using many hashtags and mentions may expose inauthentic behaviour, trolling involves broad or direct attacks or simple provocation, and is exposed through use of platform features as well as the content of posts. Patterns of activity that appeared provocative included repetitions of tweets consisting of only:

- one or more hashtags;

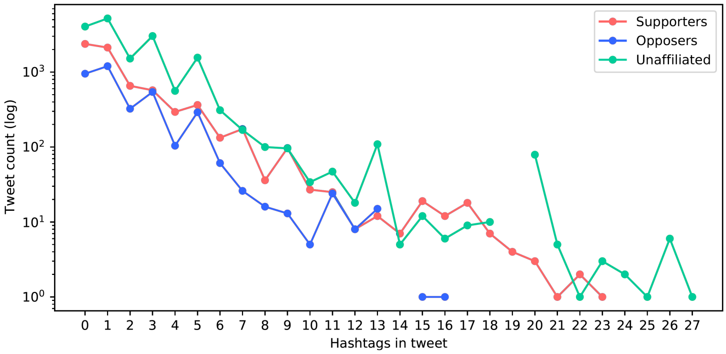

Fig. 7 The distribution of hashtag uses amongst all ArsonEmergency tweets.

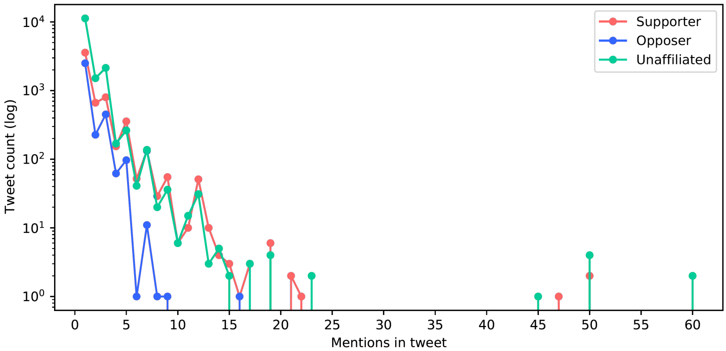

Fig. 8 The distribution of mention uses amongst all ArsonEmergency tweets.

- one or more hashtags and a trailing URL;
- one or more mentions with one or more hashtags; and
- one or more mentions with one or more hashtags and a trailing URL.

The frequencies of the occurrence of these text patterns in tweets by each group, in each phase and overall, is shown above in Table 5 (main paper). The majority of these behaviours were present in Phase 3. Although Unaffiliated accounts certainly used some of these patterns, Supporters made much more use of them, particularly more than Opposers (Figure 9). Many of the instances of hashtags followed by a URL are instances of quote tweets, where the URL is the link to the quoted tweet. These are attempts to disseminate the quoted tweet to a broader audience (engaged through the hashtags).

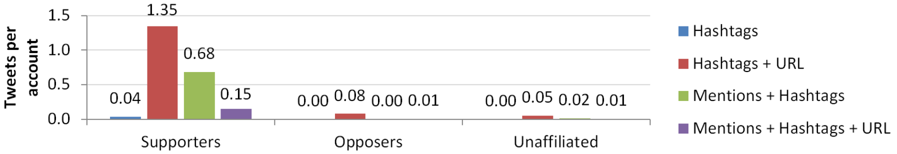

Fig. 9 Rates of use of inauthentic tweet text patterns per account for the 497 Supporters, 593 Opposers and 11,782 Unaffiliated accounts over the entire ArsonEmergency dataset.

Finally, inspection of the ten most retweeted tweet contributors revealed that three were Supporters, one was Unaffiliated, and the remainder were Opposers (including five of the top six).

5.3 Changing Names

Name switching had been observed in other discussions (Mariconti et al, 2017; Ferrara, 2017), so we examined the accounts for such behaviour. We found only 13 examples, including one Opposer and five Supporters (see Table 6). Manual inspection of the Unaffiliated, four clearly aligned with the Supporter discussion opinions and themes, based on their content, one was clearly an Opposer, and, of the remaining two, one was raising money for koalas and used hashtags to increase their reach and the other was reporting their research into the number of arson reports (referring to facts more than opinions). The behaviour of the Supporter-aligned accounts used a high proportion of retweets (12 of 18 tweets) though one of them aggressively engaged with other accounts with their six tweets. Some of the changes in screen name appeared to reflect a new 'personality' (*cf.*, Dawson and Innes, 2019), but not in a particularly deceptive way – instead, the changes of name seemed whimsical.

Table 6 Behaviour of Unaffiliated accounts that changed screen names.

Account	Inclination	Original	Reply	Retweets	Total
u_1	Supporter	2	4	0	6
u_2	Supporter	0	0	4	4
u_3	Supporter	0	0	4	4
u_4	Supporter	0	0	4	4
u_5	Opposer	1	1	0	2
u_6	Unaffiliated	4	0	0	4
u_7	Unaffiliated	2	7	2	11

6 Hashtag Use

As expected, the most prominently used hashtag for all communities was `#ArsonEmergency`, however it is clear that there are other commonly occurring hashtags. Table 7 shows the top ten hashtags used by the Supporters, Opposers and Unaffiliated in each phase, as well as the number of tweets in which they appeared.

In Phase 1, it is clear that the Supporters are trying to engage with existing climate change emergency discussion communities, as well as the media (`#7news`) and broader political discussion (`#auspol`). The few Opposer tweets seem to be poking fun at the discussion (e.g., `#RelevanceDepravationEmergency`, `#PoliticalBSEmergency`), while the Unaffiliated tweets are very broadly about the bushfires, but `#ClimateChangeHoax` is the third most used hashtag.

In the brief Phase 2, Supporters appear to be more concentrated in their promotion of the arson narrative (using `#ClimateCriminals` and `#ecoterrorism`) into the `#auspol` political discussion. Opposers seem to focus almost exclusively on using `#ArsonEmergency` rather than any other hashtags, while the Unaffiliated still follow, to some extent, the Supporters' lead with hashtags related to the arson narrative.

Finally, in Phase 3, Supporters focus mostly on just `#ArsonEmergency`, briefly linking to blaming an environmental political party and references

to hoaxes, and even reversing the attack and accusing others of being #ArsonDeniers. Opposers are firmly focused on #ArsonEmergency but start referring to an individual prominent in the media industry commonly seen as advocating against dealing with climate change. By this stage, the Unaffiliated accounts are starting to follow the Opposers' lead discussing emergency- and fire-related hashtags.

Table 7 The top ten hashtags used by the Supporters, Opposers, and Unaffiliated communities in each phase. Hashtags have been compared without considering case in the same way Twitter does. The tag $anon_1$ in Phase 3 refers to the same redacted identity in the hashtag analysis in our previous work (Weber et al, 2020), a prominent media owner.

	**Supporters**		**Opposers**		**Unaffiliated**	
	1,573 Tweets		*33 Tweets*		*1,961 Tweets*	
	Hashtag	Count	Hashtag	Count	Hashtag	Count
**Phase 1**	arsonemergency	2,086	arsonemergency	43	arsonemergency	2,534
	auspol	574	auspol	9	auspol	1,012
	climatechangehoax	232	bushfires	7	climatechangehoax	682
	climateemergency	230	tresspassemergency	6	climatechange	611
	climatechange	191	lootingemergency	6	australiaburns	307
	7news	126	bandeemergency	6	australiaburning	227
	vicfires	111	theftemergency	5	climateemergency	186
	victoria	107	relevancedepravationemergency	4	australiabushfires	142
	nswfires	90	politicalbsemergency	4	bushfireemergency	133
	globalwarming	84	denialmachine	4	australianfires	78
	121 Tweets		*327 Tweets*		*759 Tweets*	
	Hashtag	Count	Hashtag	Count	Hashtag	Count
**Phase 2**	arsonemergency	142	arsonemergency	487	arsonemergency	1,135
	auspol	79	auspol	36	auspol	194
	bushfiresaustralia	51	climateemergency	11	bushfiresaustralia	110
	climateemergency	26	scottyfrommarketing	9	climateemergency	53
	climatecriminals	23	australianbushfires	9	climatecriminals	34
	climatechange	8	australiaisburning	9	climatechange	23
	victoria	7	dontgetderailed	7	climatechangehoax	18
	ecoterrorism	6	arsonmyarse	7	scottyfrommarketing	16
	australiaisburning	6	stupidemergency	6	australianbushfires	15
	australiaburning	6	australiabushfire	6	astroturfing	15
	5,278 Tweets		*3,227 Tweets*		*14,267 Tweets*	
	Hashtag	Count	Hashtag	Count	Hashtag	Count
**Phase 3**	arsonemergency	7,731	arsonemergency	5,070	arsonemergency	21,194
	auspol	534	australiafires	649	australiafires	2,747
	climateemergency	477	climateemergency	601	climateemergency	2,566
	itsthegreensfault	270	$anon_1$	427	$anon_1$	1,778
	climatechangehoax	270	bushfires	251	australianbushfiredisaster	1,101
	climatechange	226	auspol	210	auspol	1,011
	climatehoax	220	australianbushfiredisaster	152	climatechangehoax	758
	climatecriminals	177	climatechange	140	australianbushfires	739
	bushfires	176	fakenews	137	climatechange	721
	arsondeniers	169	australianbushfires	101	bushfires	664

7 Hashtag Network Construction

We created both an account network by linking accounts that use the same hashtag, and a network of hashtags linked when used by the same account, based on the subset of tweets containing partisan hashtags described in the main paper. For accounts in the account network, u and v, which used a set of hashtags $\{h_1, h_2, ..., h_n\}$ in common, and each account x used a hashtag h with a frequency of h^x, the weight of the undirected edge $\{u, v\}$ between u and v is given by

$$w_{\{u,v\}} = \sum_{i=1}^{n} h_i^u \cdot h_i^v. \quad (2)$$

This formulation provides the maximal number of ways that u and v's hashtag uses could be combined. An alternative would be to use the minimum of h^u and h^v for all hashtags, h, as per Magelinski et al (2021)'s consideration of hashtags in their search for coordinated behaviour. In their study, however, their aim is to constrain processing requirements, while we do not have that limitation, given the size of our dataset.

The edge weights in the hashtag network were determined by the number of tweets that included both endvertex hashtags.

8 Supplementary Research Questions

Here we address a number of further research questions that we explored.

RQ1 *How can different information campaigns and the groups behind them be identified in the discussion?*

Analysis revealed two distinct polarised communities, each of which amplified particular narratives. The content posted by the most influential accounts

in each of these communities shows Supporters were responsible for the majority of arson-related content, while Opposers countered the arson narrative, debunking the errors and false statements with official information from community authorities and fact-check articles. Prior to the release of the ZDNet article, the discussion on the `#ArsonEmergency` hashtag was dominated by arson-related content. In that sense, the misinformation campaign was most effective in Phase 1, but only because its audience was small. Once the audience grew, as the hashtag received broader attention, even though Supporter activity rose dramatically, the conversation became dominated by the Opposers' narrative and related official information.

RQ2 *How did the spread of arson narrative-related misinformation and the actions of its proponents differ before and after the intervention?*

We regarded URL and hashtags as proxies for narrative and studied their dissemination, finding distinct differences between the groups and the their activity in different phases. In Phase 1, only Supporters and Unaffiliated shared URLs, the most popular of which were in the NARRATIVE category, but by the third Phase, the most popular URLs shared were DEBUNKING in nature by a ratio of 9 to 1, and NARRATIVE URLs were share only by Supporter accounts. Although it is unclear whether this change in sharing behaviour was due to changes in opinions or the influx of new accounts, there was certainly a changing of the guard. Of the 2,061 accounts active in Phase 1, less than 40% (787) remained active in Phase 3. While most Phase 1 Supporters (339 of 360) posted in Phase 3, many fewer Unaffiliated accounts did (427 of 1,680) indicating that the Supporters lost the support of most of the Phase 1 Unaffiliated accounts.

The diversity of URL and hashtag use also changed from Phase 1 to Phase 3: while the number of active Supporters grew modestly from 360 to 474, the

number of unique external URLs they used grew more, proportionately, from 193 to 321. Opposers and Unaffiliated used more unique URLs in Phase 3 (492 and 4,368, respectively), but Figures 12 and 16 (in the main paper) show they focused on a small set of URLs more than Supporters did.

The number of hashtags Supporters used increased from 191 hashtags used 5,382 times to 543 hashtags used 14,472 times. This implies Supporters attempted to connect `#ArsonEmergency` with other hashtag-based communities, which could have been to in order to promote their message widely, to co-opt existing discussion spaces, or due to non-Australian contributors being unfamiliar with which hashtags would be relevant to the mostly Australian audience. From Phase 1 to Phase 3, Opposer activity increased from 34 hashtags used 150 times to 200 hashtags used 9,549 times, and the hashtag network visualisation in Figure 4b (in Weber et al, 2020) confirms Opposers focused the majority of their discussion on a comparatively small number of hashtags.

The `#ArsonEmergency` discussion's growth rate was similar to another contemporary discussion (the `#AustraliaFire` campaign), inasumuch as they both experienced events causing significant changes in their participation, but it was clearly different from that of a well-established discussion (`#Brexit`).

RQ3 *To what degree did the polarised groups receive support from outside Australia?*

Based on manual inspection of accounts' free text 'location' fields, the Supporter group included more non-Australian than Opposers, with the greatest number of non-Australian accounts Unaffiliated with either, but the vast majority of all groups indicated they were located in Australia ($> 70\%$). Despite the large number of Unaffiliated accounts present in Phase 1 (1,680), the majority joined the discussion in Phase 3, likely bringing in the majority of non-Australian accounts. Investigations of content dissemination also revealed

that Opposers received the majority of Unaffiliated support, resulting in a majority of debunking article shares in Phase 3 from a majority of narrative-aligned article shares in Phase 1, so it is possible that this also included non-Australian support. Given most accounts do not report their location, and locations have not been verified, this conclusion remains speculative.

References

Assenmacher D, Weber D, Preuss M, et al (2021) Benchmarking crisis in social media analytics: A solution for the data-sharing problem. Social Science Computer Review p 089443932110122. https://doi.org/10.1177/08944393211012268

Cresci S (2020) A decade of social bot detection. Communications of the ACM 63(10):72–83. https://doi.org/10.1145/3409116

Dawson A, Innes M (2019) How Russia's Internet Research Agency built its disinformation campaign. The Political Quarterly 90(2):245–256. https://doi.org/10.1111/1467-923x.12690

Feng S, Wan H, Wang N, et al (2021) TwiBot-20: A comprehensive Twitter bot detection benchmark. In: Proceedings of the 30th ACM International Conference on Information & Knowledge Management. ACM, CIKM '21, pp 4485–4494, https://doi.org/10.1145/3459637.3482019

Ferrara E (2017) Disinformation and social bot operations in the run up to the 2017 French presidential election. First Monday 22(8). https://doi.org/10.5210/fm.v22i8.8005

Graham T, Keller TR (2020) Bushfires, bots and arson claims: Australia flung in the global disinformation spotlight. The Conversation URL https://thec

onversation.com/bushfires-bots-and-arson-claims-australia-flung-in-the-global-disinformation-spotlight-129556

Keller T, Graham T, Angus D, et al (2020) 'Coordinated Inauthentic Behaviour' and other online influence operations in social media spaces. Presented at the Annual Conference of the Association of Internet Researchers, AoIR 2020, URL https://spir.aoir.org/ojs/index.php/spir/article/view/11132/9763

Kumar S, Hamilton WL, Leskovec J, et al (2018) Community interaction and conflict on the Web. In: WWW. ACM, pp 933–943, https://doi.org/10.1145/3178876.3186141

Leetaru K (2019) Twitter users mostly retweet politicians and celebrities. That's a big change. The Washington Post, URL https://www.washingtonpost.com/politics/2019/03/08/twitter-users-mostly-retweet-politicians-celebrities-thats-big-change/

Magelinski T, Ng LHX, Carley KM (2021) A synchronized action framework for responsible detection of coordination on social media. CoRR abs/2105.07454. https://arxiv.org/abs/2105.07454

Mariconti E, Onaolapo J, Ahmad SS, et al (2017) What's in a name?: Understanding profile name reuse on Twitter. In: Proceedings of the 26th International Conference on World Wide Web. ACM, WWW '17, pp 1161–1170, https://doi.org/10.1145/3038912.3052589

Mariconti E, Suarez-Tangil G, Blackburn J, et al (2019) "You know what to do": Proactive detection of YouTube videos targeted by coordinated hate attacks. PACMHCI 3(CSCW):207:1–207:21. https://doi.org/10.1145/3359309

Shao C, Ciampaglia GL, Flammini A, et al (2016) Hoaxy: A platform for tracking online misinformation. In: WWW (Companion Volume). ACM, pp 745–750, https://doi.org/10.1145/2872518.2890098

Starbird K, Wilson T (2020) Cross-Platform Disinformation Campaigns: Lessons Learned and Next Steps. Harvard Kennedy School Misinformation Review https://doi.org/10.37016/mr-2020-002

Stilgherrian (2020) Twitter bots and trolls promote conspiracy theories about Australian bushfires. ZDNet, URL https://www.zdnet.com/article/twitter-bots-and-trolls-promote-conspiracy-theories-about-australian-bushfires/

The Soufan Center (2021) Quantifying the Q Conspiracy: A Data-Driven Approach to Understanding the Threat Posed by QAnon. Special report, The Soufan Center, URL https://thesoufancenter.org/research/quantifying-the-q-conspiracy-a-data-driven-approach-to-understanding-the-threat-posed-by-qanon/

Weber D, Nasim M, Falzon L, et al (2020) #ArsonEmergency and Australia's "Black Summer": Polarisation and misinformation on social media. Lecture Notes in Computer Science (LNCS) pp 159–173. https://doi.org/10.1007/978-3-030-61841-$4_1$1

Weedon J, Nuland W, Stamos A (2017) Information operations and Facebook. White Paper, Facebook, URL https://fbnewsroomus.files.wordpress.com/2017/04/facebook-and-information-operations-v1.pdf
